# Comprehensive metabolic profiling of Parkinson’s disease by liquid chromatography-mass spectrometry

**DOI:** 10.1186/s13024-021-00425-8

**Published:** 2021-01-23

**Authors:** Yaping Shao, Tianbai Li, Zheyi Liu, Xiaolin Wang, Xiaojiao Xu, Song Li, Guowang Xu, Weidong Le

**Affiliations:** 1 Center for Clinical Research on Neurological Diseases, The First Affiliated Hospital, Dalian Medical University, 193 Lianhe Road, Dalian, China; 2 Liaoning Provincial Key Laboratory for Research on the Pathogenic Mechanisms of Neurological Diseases, The First Affiliated Hospital, Dalian Medical University, 193 Lianhe Road, Dalian, China; 3 CAS Key Laboratory of Separation Science for Analytical Chemistry, Dalian Institute of Chemical Physics, Chinese Academy of Sciences, 457 Zhongshan Road, Dalian, 116023 China; 4 Institute of Neurology, Sichuan Academy of Medical Science-Sichuan Provincial Hospital, Medical School of UESTC, Sichuan, China

**Keywords:** Parkinson’s disease, Metabolomics, Biomarker, Metabolic disturbances, Bile acid profile

## Abstract

**Background:**

Parkinson’s disease (PD) is a prevalent neurological disease in the elderly with increasing morbidity and mortality. Despite enormous efforts, rapid and accurate diagnosis of PD is still compromised. Metabolomics defines the final readout of genome-environment interactions through the analysis of the entire metabolic profile in biological matrices. Recently, unbiased metabolic profiling of human sample has been initiated to identify novel PD metabolic biomarkers and dysfunctional metabolic pathways, however, it remains a challenge to define reliable biomarker(s) for clinical use.

**Methods:**

We presented a comprehensive metabolic evaluation for identifying crucial metabolic disturbances in PD using liquid chromatography-high resolution mass spectrometry-based metabolomics approach. Plasma samples from 3 independent cohorts (*n* = 460, 223 PD, 169 healthy controls (HCs) and 68 PD-unrelated neurological disease controls) were collected for the characterization of metabolic changes resulted from PD, antiparkinsonian treatment and potential interferences of other diseases. Unbiased multivariate and univariate analyses were performed to determine the most promising metabolic signatures from all metabolomic datasets. Multiple linear regressions were applied to investigate the associations of metabolites with age, duration time and stage of PD. The combinational biomarker model established by binary logistic regression analysis was validated by 3 cohorts.

**Results:**

A list of metabolites including amino acids, acylcarnitines, organic acids, steroids, amides, and lipids from human plasma of 3 cohorts were identified. Compared with HC, we observed significant reductions of fatty acids (FFAs) and caffeine metabolites, elevations of bile acids and microbiota-derived deleterious metabolites, and alterations in steroid hormones in drug-naïve PD. Additionally, we found that L-dopa treatment could affect plasma metabolome involved in phenylalanine and tyrosine metabolism and alleviate the elevations of bile acids in PD. Finally, a metabolite panel of 4 biomarker candidates, including FFA 10:0, FFA 12:0, indolelactic acid and phenylacetyl-glutamine was identified based on comprehensive discovery and validation workflow. This panel showed favorable discriminating power for PD.

**Conclusions:**

This study may help improve our understanding of PD etiopathogenesis and facilitate target screening for therapeutic intervention. The metabolite panel identified in this study may provide novel approach for the clinical diagnosis of PD in the future.

**Supplementary Information:**

The online version contains supplementary material available at 10.1186/s13024-021-00425-8.

## Background

Parkinson’s disease (PD) is the most prevailing movement disorder and represents the second most common neurodegenerative disease, affecting approximately 1% of the population above 60 years [1, 2]. The main neuropathological characteristics of PD are marked loss of dopaminergic neurons within substantia nigra and the presence of intracytoplasmic α-synuclein-containing Lewy bodies, manifesting as reduced facilitation of voluntary movement [2, 3]. The current diagnosis of PD essentially relies on evaluation of clinical signs. Although neuroimaging technologies have improved the diagnosis and staging of PD, these detections are expensive and labor intensive [4, 5]. Therefore, many studies have been dedicated to the discovery of biomarkers that may assist the diagnosis of PD. However, no peripheral blood derived biomarkers have been used clinically at present [6–8]. Accumulated evidence indicated that PD is multifactorial; a combination of age, genetics and environmental factors might contribute to its onset and progression [9, 10].

Emerging evidence indicated that peripheral alterations including metabolic dysregulations might precede and contribute to neurodegeneration [11–14]. Deciphering the molecular networks that distinguish PD from healthy individuals and patients with other PD-unrelated diseases might lead to novel insight into PD pathogenesis and the identification of crucial biomarkers. Liquid chromatography-mass spectrometry- (LC-MS) based metabolomics is a powerful tool to profile metabolite changes. It has been utilized to decipher metabolic reprogramming in many types of disease including neurodegenerative disorders [12, 15, 16]. Previous studies indicated the involvement of oxidative stress and dyshomeostasis in metabolism of catecholamine, tryptophan and caffeine in PD [3, 16, 17]. Several potential biomarkers have been identified, such as acylcarnitines [18], quinolinic acid (QA)/kynurenic acid (KA) ratio [19], N8-acetyl spermidine and lipids [10, 20]. However, many studies cannot corroborate each other, possibly due to the limited sample size, lack of validation cohort and confounding factors from clinical heterogeneity, analytical methodology and antiparkinsonian medication. Recently, bile acid (BA) metabolism has been linked to liver diseases, diabetes, inflammatory bowel diseases and neurodegenerative disorders [12, 21, 22]. In addition to their important roles in lipid digestion and absorption, BAs act as signaling molecules by activating membrane and nuclear receptors as well as ion channels [23]. However, to the best of our knowledge, there has been no systematic study on the profiling of BAs in PD population.

Herein, we utilized a LC-MS based untargeted metabolomics approach to investigate the metabolic changes associated with PD in 3 well-characterized cohorts. Using a method of targeted extraction and integration of the chromatographic peak, we also presented a comprehensive analysis of BA profiles in PD. Additionally, the influences from various variables (gender, age, duration, stage, and pharmacological treatment) to the level of metabolites were also investigated. We aimed to identify the most promising metabolic biomarkers for the diagnosis of PD, and corresponding metabolic pathways that might contribute to a better understanding of the biochemical impairments involved in the disease.

## Methods

### Participants

Totally, 460 plasma samples including 223 from PD, 169 from healthy controls (HCs) and 68 from patients with PD-unrelated neurological diseases were enrolled at the First Affiliated Hospital of Dalian Medical University. PD patients were diagnosed by at least two experienced neurologists based on the Movement Disorder Society Clinical Diagnostic Criteria for Parkinson’s disease, and their favorite response to L-dopa therapy [24]. HC subjects were recruited from the Health Examination Center. In cohort 1, all the PD patients were drug-naïve. In cohort 2, 97 individuals were included, of which 51 were treated and 14 were drug-naïve, and 32 were HC. In cohort 3, apart from PD and HC, a matched PD-unrelated neurological disease control (NDC) group comprised of 27 cerebrovascular diseases, 9 epilepsy, 9 peripheral vertigo, 8 peripheral neuropathy, 8 anxiety/sleep disorders, 5 syncope and 2 myasthenia gravis were included. Most of the patients in NDC did not receive regular medications, except for epilepsy patients who were routinely treated with antiepileptic drugs. Detailed descriptions of the participants are given in Table 1. All subjects or their legally authorized caregivers provided informed consents and this study was approved by the Ethics Committee of the hospital.

**Table 1:** Study population features

	PD(***n*** = 223)	HC (***n*** = 169)	NDC (***n*** = 68)	***p*** value ^**a**^
**Cohort 1**				
Number of individuals	36	43	–	
Age, mean ± SE	64.4 ± 1.5	65.5 ± 1.2	–	0.6826
Gender (m/f)	20/16	25/18	–	0.8173
Duration of disease (year), mean ± SE	4.4 ± 0.8	–	–	
H&Y stage, mean ± SE	2.1 ± 0.1	–	–	
**Cohort 2**				
Number of individuals	65	32	–	
Age, mean ± SE	66.2 ± 1.3	64.6 ± 1.7	–	0.6367
Gender (m/f)	36/29	18/14	–	0.9651
Duration of disease (year), mean ± SE	5.1 ± 0.5	–	–	
H&Y stage, mean ± SE	2.1 ± 0.1	–	–	
**Cohort 3**				
Number of individuals	122	94	68	
Age, mean ± SE	68.2 ± 1.0	68.6 ± 0.8	68.6 ± 1.1	0.9576
Gender (m/f)	68/54	51/43	37/31	0.9720
Duration of disease (year), mean ± SE	5.6 ± 0.4	–	–	
H&Y stage, mean ± SE	2.4 ± 0.1	–	–	

^a^ The Mann–Whitney U test (PD and HC) or one-way ANOVA (PD, HC and NDC) was used to calculate the statistical significance difference in age distribution between the groups in each cohort. A chi-square test was applied to investigate the difference in gender composition

In cohort1, all the PD patients were drug-naïve patients. In cohort 2, 14 were drug-naïve, 51 were treated PD patients (L-dopa-treated, pramipexol-treated or the combination of L-dopa and pramipexol-treated). In cohort 3, 27 were drug-naïve, 95 were treated PD patients. Most of the patients in NDC group did not receive regular medications, except for 9 patients with epilepsy who were treated with antiepileptic drugs. There is no significant difference in caffeine consumption between PD and controls. m/f indicates ratio of the number of males to the number of females

### Biospecimen collection and processing

Fasting venous blood samples were collected into ethylene diamine tetra-acetic acid containing vacutainers (Insepack, SEKISUI medical technology) by direct venipuncture. Subsequently, plasma samples were transferred into sterile tubes after centrifugation at 3000 rpm for 5 min and stored at − 80 °C. Before metabolomics analysis, plasma samples were visually checked for hemolysis, and there were no hemolytic specimens used in our study. Plasma samples were prepared as previously described with slight modifications [25]. 130 μL of plasma was deproteinized with 4 volumes of methanol containing internal standards (ISs, **Table S****1**). After centrifugation at 13,000 g for 10 min, the resulting supernatant was divided into two aliquots and lyophilized. The dried samples were reconstituted in 65 μL of methanol/water (1/3) and analyzed by LC-MS operated in ESI positive (ESI+, basic species) and negative (ESI-, acidic species) modes. To evaluate the repeatability of sample pretreatment and monitor the stability of instrument analysis, quality control (QC) samples were made by mixing equal amounts of each sample, prepared identically to the analytical sample, and analyzed after 10 sample runs. Additionally, blank samples with ultrapure water instead of plasma were made and treated with the same method and analyzed before the sequence was run to assess potential background interference during the experimental process.

### LC-MS analysis

Metabolic profiling was performed on an Ultra Performance Liquid Chromatography (UPLC, Waters, Manchester, UK) coupled with tripleTOF™ 5600 plus (Applied Biosystems, Foster City, CA) MS system. In ESI+ mode, extracts were retained and gradient eluted from an ACQUITY UPLC BEH C8 column using water and acetonitrile with 0.1% formic acid solution. In ESI- mode, extracts were retained and gradient eluted from an ACQUITY UPLC HSS T3 column using water and 95% methanol containing 6.5 mM ammonium bicarbonate. Detailed chromatographic and MS conditions are given in **Supplementary Materials****.** Instrument control and data acquisition were conducted using Analyst TF 1.7 software.

### Raw data preprocessing

Total ion chromatograms were analyzed using Peakview (version 1.2.0.3, Applied Biosystems). The acquired raw data were imported into Marker View (version 1.2.1.1, Applied Biosystems) for peak extraction and alignment. Features that detected in at least 80% of acquired samples in disease or control group were retained [26]. Besides, due to the automatic peak alignment process, the information of low abundance of metabolites may be lost. To obtain a comprehensive and accurate profile of BA, we performed a method of targeted extraction and integration of the chromatographic peak using MultiQuant (version 2.1, Applied Biosystems) software (**Supplementary Materials**). Prior to statistical analysis, original datasets were calibrated by ISs. Each ion feature in QC sample was calibrated with all ISs and the relative standard deviation (RSD) after each calibration was calculated. We used IS that can achieve the minimum RSD in QC sample to calibrate the ion features in analytical samples. For metabolites identified in 3 datasets, the original peak areas were calibrated using identical ISs.

### Metabolite identification

The processing of metabolite identification was carried out with the OSI/SMMS software [27]. In brief, the retention time of each ion feature was calibrated using ISs to estimate fluctuations between batches. The calibrated retention time and MS data of ion features in analyzed samples were searched against an in-house database by comparing the qualitative information of each metabolite with that of reference chemical standards. Comprehensive procedures including precursor ion alignment and ion fusion, database searching and scoring were applied to remove the artifacts and background noise [27].

### Statistics

SIMCA (version 13.0.0.0., Umetrics AB, Umea, Sweden) was used to perform multivariate analysis including principal component analysis (PCA), partial least square discriminant analysis (PLS-DA) and orthogonal PLS-DA (OPLS-DA). Permutation test was conducted to avoid a potential risk of overfitting. We used the Mann-Whitney U test to calculate the statistical significance. A standard Benjamini-Hochberg method was applied to control the false discovery rate (FDR) for multiple hypothesis testing [28]. Linear regressions were applied to investigate associations of metabolites with age, duration and disease severity using PASW Statistics (version 18.0.0). Hierarchical cluster analysis was performed using the MeV software package (version 4.8.1). Binary logistic regression analysis was applied to generate a mathematical model for PD discrimination. The predictive performance of the model was assessed by estimating the area under the receiver operating characteristic (ROC) curve (AUC), which is commonly used to evaluate overall discriminant ability [29].

## Results

**Fig. 1:**
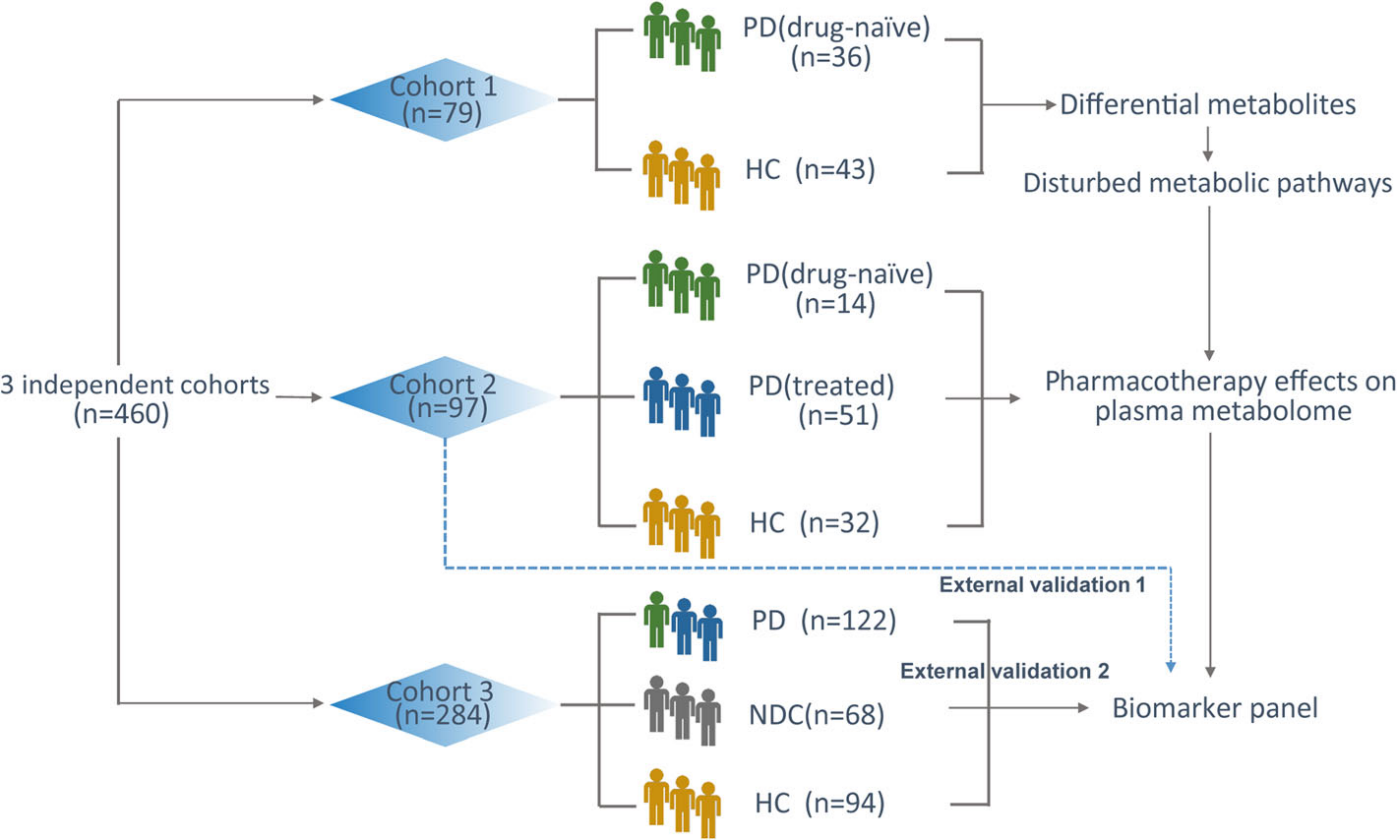
Flow chart of the experimental design. Totally, 460 plasma samples were collected and divided into three independent data sets for metabolomics analysis

The demographic and clinical characteristics of the involved subjects are presented in Table 1. A total of 460 individuals were enrolled and divided into 3 independent cohorts (Fig. 1). There was no significant difference in age distribution or gender composition between the groups in each cohort, which indicated that the subjects in each group were comparable.

**Fig. 2:**
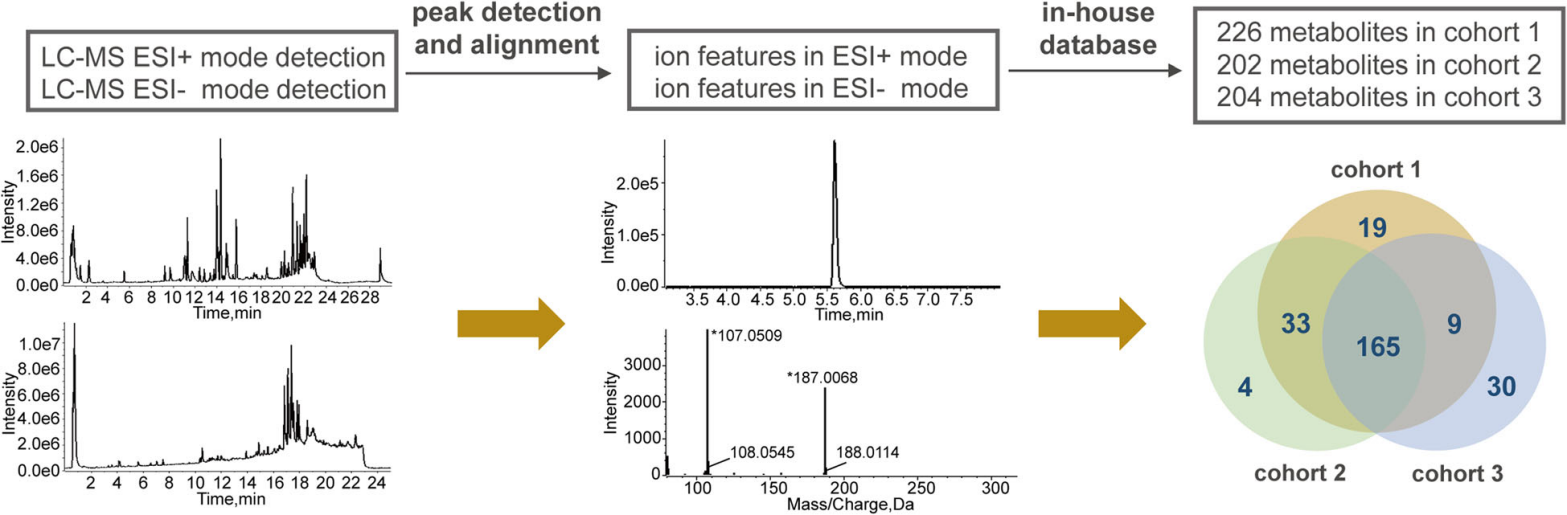
Overview of the detection and identification of metabolites in three cohorts. Briefly, each plasma sample was analyzed by both LC-MS ESI+ mode and ESI- mode to facilitate the ionization and detection of alkaline compounds and acidic compounds, respectively. After peak detection and alignment and metabolite recognition, 226, 202 and 204 metabolites were identified in each cohort

After instrumental analysis, peak detection and alignment, and metabolite recognition, 226, 202 and 204 metabolites were identified in cohort 1, cohort 2 and cohort 3, respectively (Fig. 2). The data matrices of the identified metabolites were used for the following statistical analysis. We first assessed the metabolomic data quality of the datasets drawn from 3 independent cohorts. As shown in **Figure S****1****a-1c,** the QC samples clustered in the center on the PCA score plots, suggesting that the analyses were repeatable and robust. Then we further analyzed the RSD of metabolites in QC samples (**Figure S****1****d-1f)**, 90.3, 99.5 and 98.5% of the metabolites had RSD values < 30% in each cohort, which further confirmed the reliability of the data.

Considering that the detections of fatty acids (FFAs) might be interfered with residuals from plastic containers [30], we further analyzed and compared the contents of FFAs in blank (ultrapure water) and analytical samples (QC samples). Totally, 35 FFAs were detected in plasma samples. Among them, 8 FFAs were not detectable in blank sample. For the rest of 27 FFAs, the contents of 19 FFAs in the blank were less than 10% of the analytical sample. We found that the contents of only 2 FFAs were more than 30% of the analytical sample (**Table S****2**). Besides, we used stable isotope labeled FFAs as IS to calibrate the original raw data to reduce systematic errors drawn from sample pretreatment and instrumental analysis processes. After IS calibration, 94% FFAs had RSDs of less than 15% and all the FFAs had RSDs of less than 30% in QC samples (**Table S****2**), which implied that the interference derived from plastic containers did not significantly affect the detection reproducibility and relative quantification of FFA in this study.

### Metabolic signatures of drug-naïve PD

A comparative study involving drug-naïve PD (DN-PD) and matched HC was initially performed to investigate the metabolic signatures of PD and rule out potentially confounding effects of symptomatic medications. The metabolic differences between males and females in DN-PD and HC were firstly investigated. As shown in **Figure S****2**, there was no clear separation between males and females in both HC and DN-PD on the PCA score plot. By performing univariate analysis with FDR calibration, 24 metabolites including acetylcholine, creatinine, several amino acids, phosphatidylcholines (PCs) and sphingomyelins (SMs) were found different between genders in HC (**Table S****3**). However, no gender difference was found in DN-PD. These findings suggested that the metabolic profile of PD was significantly altered compared to that of healthy individuals.

**Fig. 3:**
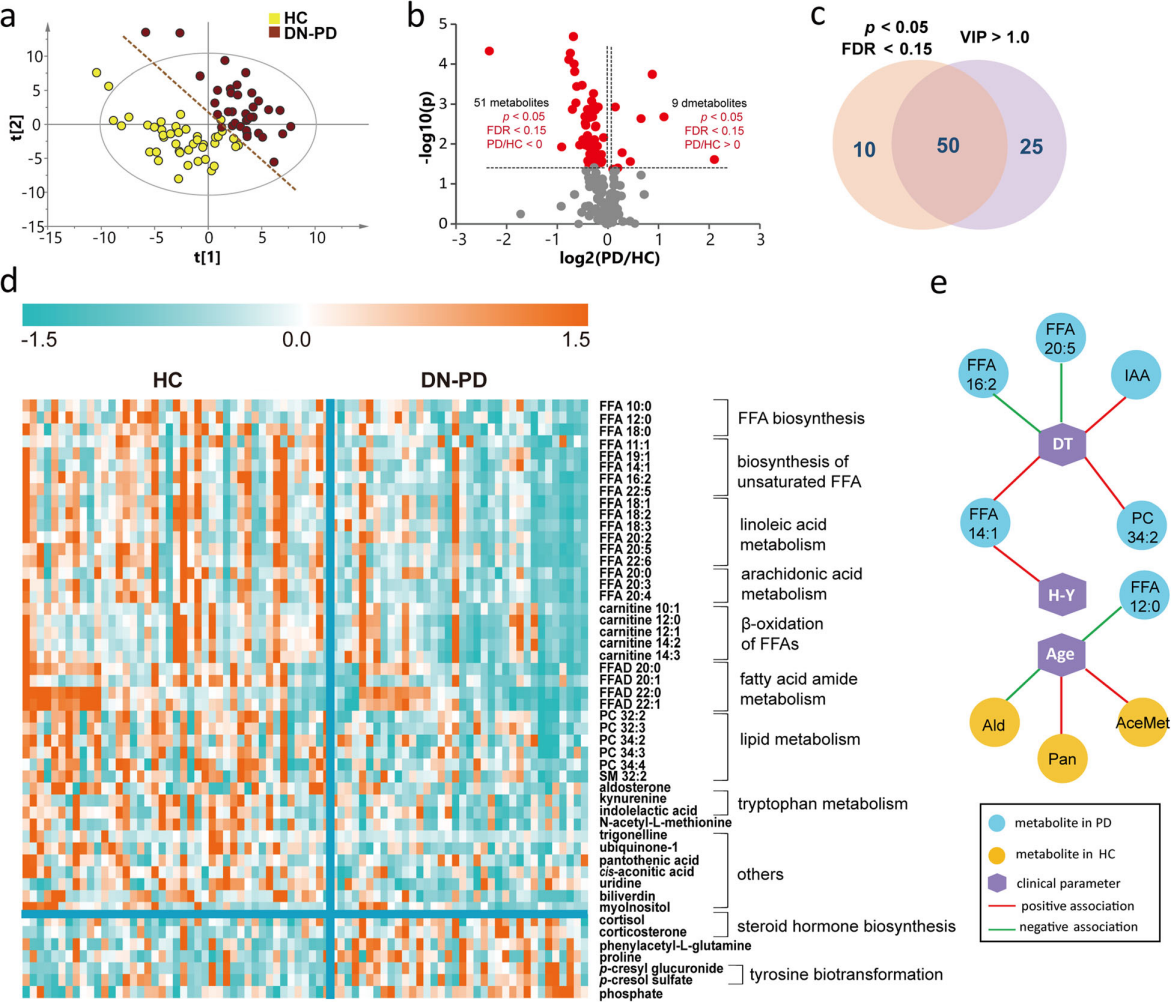
Altered metabolic profiles in drug-naïve PD compared with HC. **a** PLS-DA score plot of DN-PD and HC in cohort 1. R2X = 0.212, R2Y = 0.758, Q2 = 0.594. The analysis of variance was based on cross-validated prediction residuals (CV-ANOVA) data: *p* = 9.6139E-014, F factor = 26.7611. **b** Volcano plot of the differential metabolites in DN-PD filtered by univariate analysis. **c** Venn plot of the differential metabolites filtered by PLS-DA model and univariate analysis. **d** Heat map of the 50 differential metabolites in DN-PD. Blue indicates a decreased level, orange indicates an increased level. **e** Associations of metabolites with disease severity, duration time and age. DT: duration time; IAA: indolelactic acid; Ald: aldosterone; Pan: pantothenic acid; AceMet: N-acetyl-L-methionine

To maximize identification of differential metabolites in PD patients, we constructed a PLS-DA model. The plasma metabolome of DN-PD was clearly separated from HC on the score plot (Fig. 3a). Permutation test was performed to ensure that the model was not overfitted (**Figure S****3****a**). Of the 226 metabolites, 75 metabolites contributed significantly to the distinction of DN-PD and HC with VIP values > 1. We further performed a Mann-Whitney U test with FDR calibration and found 60 metabolites were significantly changed (Fig. 3b). The overlapped 50 metabolites were considered as differential metabolites in PD (Fig. 3c). Of these, levels of 43 metabolites were decreased, including acyl carnitines, PCs, FFAs, FFA amides (FFADs), indolelactic acid, trigonelline and kynurenine, among others. Conversely, levels of 7 metabolites including phenylacetyl-L-glutamine, *p*-cresol glucuronide, *p*-cresol sulfate, proline, cortisol, corticosterone, and phosphate were significantly increased in PD (Fig. 3d**, Table S****4**). Based on these differential metabolites identified in PD, we further carried out pathway analysis and found that metabolic dysregulations in unsaturated FFAs biosynthesis (especially linoleic acid, linolenic acid, and arachidonic acid metabolism), steroid hormone biosynthesis, pantothenate and CoA biosynthesis and amino acids metabolism might be involved in PD etiopathogenesis (**Figure S****4**).

Then, we investigated the associations of the differential metabolites with disease severity, duration, and age. The disease severity of PD was assessed based on Modified Hoehn - Yahr (H-Y) staging [31]. We found that FFA 14:1 was positively associated with disease severity (Fig. 3e**, Table S****5**). Levels of FFA 14:1, PC 34:2 and indolelactic acid were positively associated with the duration time of the disease, whereas FFA 20:5 and FFA 16:2 were negatively associated (Fig. 3e**, Table S****6**). Interestingly, metabolites in HC and PD showed different age associations. Aldosterone, pantothenic acid, and N-acetyl-L-methionine were associated with age in HC, however, only 1 metabolite FFA 12:0 showed association with age in PD (Fig. 3e**, Table S****7**). These findings provided further evidence that HC and PD had different metabolic patterns.

### Impacts of drug therapies on plasma metabolome of PD

Generally, PD patients are usually treated with different types of antiparkinsonian medication. To assess the possible drug-induced changes in plasma metabolome, we recruited an independent population including DN-PD, treated PD (L-dopa-treated PD (DO-PD), pramipexole-treated PD (PR-PD) and the combination of L-dopa and pramipexole-treated PD (CO-PD)) and HC for metabolomics analysis.

**Fig. 4:**
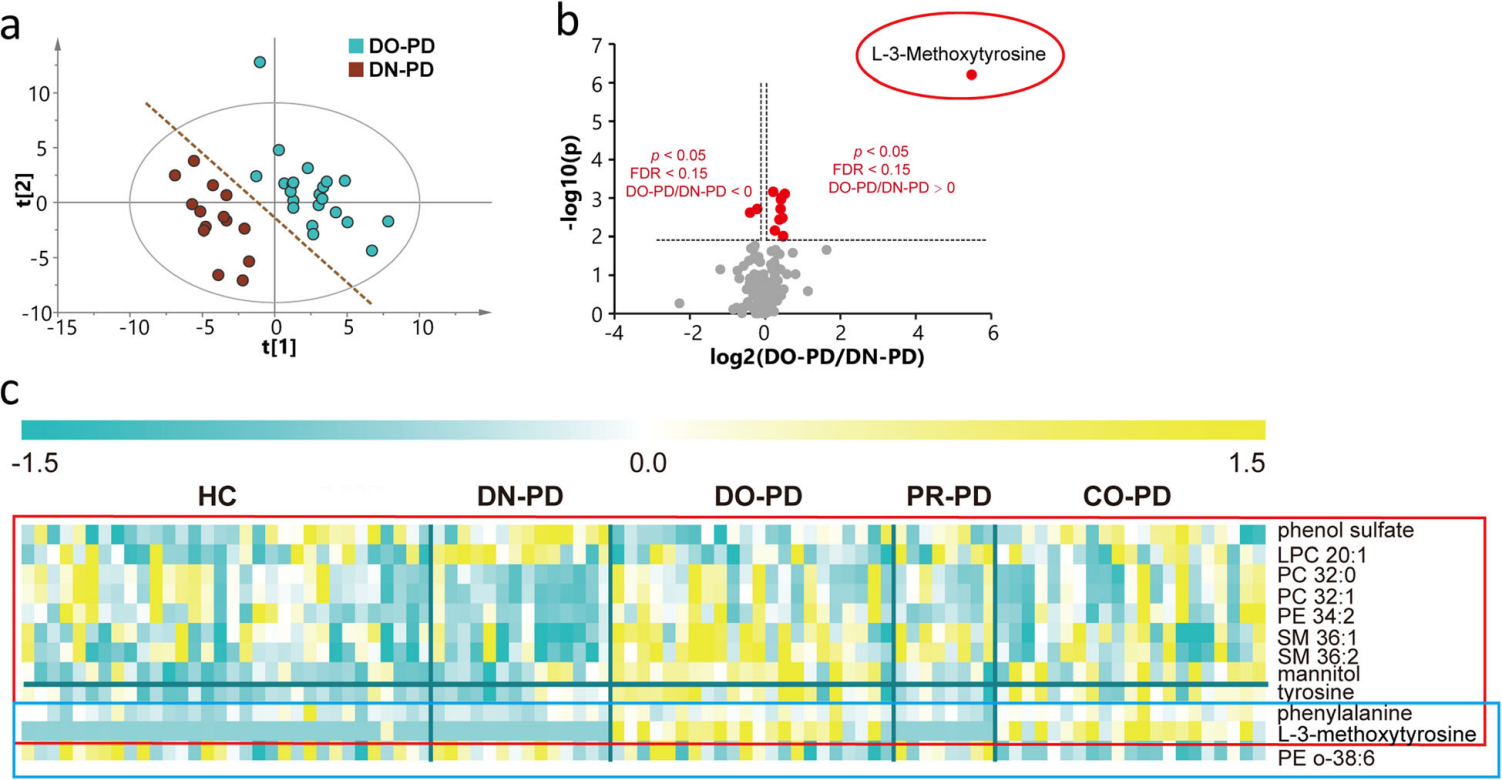
Impacts of drug therapies on plasma metabolome of PD. **a** PLS-DA score plot of DN-PD and DO-PD in cohort 2. R2X = 0.179, R2Y = 0.863, Q2 = 0.323. CV-ANOVA data: *p* = 0.0030, F factor = 5.0488. **b** Volcano plot of the significantly changed metabolites in the plasma of PD patients after L-dopa treatment. **c** Heat map of differential metabolites between treated PD and DN-PD, which showed possible drug effects to the plasma metabolome

As shown in **Figure S****1****b**, the plasma metabolome of PD showed a trend of separation from HC, whereas different subgroups of PD displayed partial overlap. To investigate the impacts of medications on plasma metabolome, we constructed PLS-DA models between DN-PD and different types of treated PD. We found that the metabolome of DN-PD showed a difference from that of DO-PD on the score plot of PLS-DA model without overfitting (Fig. 4a**, Figure S****3****b**). However, PLS-DA models between DN-PD and PR-PD or CO-PD did not show clear separation. These findings implied that L-dopa treatment could cause significant impact on the plasma metabolome. However, neither pramipexole treatment nor the combination of L-dopa and pramipexole treatment could cause significant impacts. Based on multivariate and univariate statistical analyses (VIP value, *p* value and FDR), 11 metabolites including phenol sulphate, lysophosphatidylcholine (LPC), PC, SM, L-3-methoxytrosine, phenylalanine, etc. were found relevant to L-dopa treatment. We found that L-dopa treatment resulted in a remarkable increase in the level of L-3-methoxytyrosine, which is a major metabolite of L-dopa (Fig. 4b). Based on univariate statistical analysis solely, no metabolites were found relevant to pramipexole treatment alone, and 3 metabolites including phenylalanine, L-3-methoxytyrosine and PE o-38:6 were related to combinational treatment (Fig. 4c).

### BA profiles in PD patients

We demonstrated a significant elevation of liver-derived primary bile acid CA, and bacterially generated secondary bile acid DCA as well as conjugated bile acid TDCA, GDCA, GDCS, TCAS and GLCAS in PD compared to HC. Notably, both L-dopa and combinational treatments could alleviate the elevations of BAs in PD patients. However, no significant alterations were found after pramipexole treatment alone (Table 2). Interestingly, UDCA and TUDCA, which were reported neuroprotective in PD [32], were decreased in the plasma of PD patients, although there was no statistical significance. By calculating the ratios of BAs, we assessed the activities of enzymes involved in BA synthesis and found a significant elevation of CA/CDCA in PD, indicating a shift in initial cholesterol metabolism from alternative pathway to primary pathway. Besides, most of the ratios were increased in DN-PD compared to HC, the elevations of these BA ratios tend to be alleviated by medication.

**Table 2:** Statistical results of bile acids contents and ratios in drug-naïve PD, treated PD and healthy controls in cohort 1 and cohort 2

Category	BAs/ratios	Cohort 1			Cohort 2								
		DN-PD vs. HC			DN-PD vs. HC			DO-PD vs. DN-PD			CO-PD vs. DN-PD		
		***p***	FDR	FC_**1**_	***p***	FDR	FC_**2**_	***p***	FDR	FC_**3**_	***p***	FDR	FC_**4**_
Primary	**CA**	**0.0183**	**0.1034**	**2.64**	0.4102	0.6766	**1.98**	0.9096	0.9096	0.83	0.9329	0.9912	1.09
	CDCA	0.1978	0.3422	1.53	0.4378	0.6766	1.48	0.5702	0.7455	0.77	0.8531	0.9912	0.94
Secondary	**DCA**	**0.0033**	**0.0499**	**1.76**	0.7114	0.7802	**1.02**	0.2237	0.6337	0.81	0.3545	0.7532	0.90
	UDCA	0.2595	0.4201	0.67	0.9905	0.9905	0.92	0.0770	0.2908	0.43	0.6494	0.9912	0.95
	LCA	0.4026	0.6029	1.19	0.7837	0.8074	1.03	0.3384	0.6907	0.67	0.9866	1.0000	0.87
Primary conjugated	**GCA**	**0.0424**	**0.1601**	**1.43**	**0.0272**	**0.2314**	**1.72**	**0.0055**	**0.0376**	**0.40**	0.0529	0.3594	0.81
	TCA	0.1776	0.3422	1.31	**0.0226**	**0.2314**	**1.99**	**0.0017**	**0.0197**	**0.29**	**0.0090**	**0.1872**	**0.71**
	**TCAS**	**0.0073**	**0.0499**	**2.07**	**0.0412**	**0.2804**	**2.78**	0.4266	0.6907	0.57	0.0665	0.3768	0.37
	TCDCA	0.4492	0.6109	**1.47**	**0.0054**	**0.1230**	**2.05**	**0.0067**	**0.0382**	**0.36**	**0.0110**	**0.1873**	**0.47**
	GCDCA	0.1034	0.2510	**1.44**	**0.0072**	**0.1230**	**1.75**	**0.0010**	**0.0197**	**0.43**	**0.0299**	**0.3385**	**0.56**
	GCDCS	0.9960	0.9960	0.99	0.4239	0.6766	1.08	**0.0027**	**0.0228**	**0.43**	0.1727	0.5337	0.68
Secondary conjugated	**TDCA**	**0.0069**	**0.0499**	**2.52**	0.0546	0.3096	**3.63**	0.3551	0.6907	0.37	0.0829	0.4027	0.41
	**GDCA**	**0.0006**	**0.0196**	**2.81**	0.0643	0.3122	**2.94**	0.3384	0.6907	0.46	0.1523	0.5337	0.55
	TUDCA	0.7675	0.8417	0.89	0.2373	0.5379	1.11	**0.0012**	**0.0197**	**0.41**	**0.0416**	**0.3540**	**0.67**
	GUDCA	0.6774	0.7942	1.02	0.0878	0.3318	1.35	**0.0119**	**0.0578**	**0.38**	0.2320	0.6066	0.58
	**GDCS**	**0.0278**	**0.1182**	**1.63**	0.2101	0.5379	**2.43**	0.3898	0.6907	0.50	0.4489	0.8475	0.51
	TLCA	0.1744	0.3422	1.22	0.1421	0.4391	2.00	0.1398	0.4754	0.49	0.2738	0.6206	0.68
	TLCAS	0.0689	0.1952	1.39	0.2670	0.5673	2.11	0.3551	0.6907	0.67	0.2738	0.6206	0.43
	GLCA	0.1909	0.3422	0.98	0.3967	0.6766	0.99	0.4854	0.7455	0.92	0.9062	0.9912	1.00
	TDCS	0.7598	0.8417	1.47	0.228	0.5379	1.41	**0.0496**	**0.2109**	**0.49**	0.1623	0.5337	0.57
	**GLCAS**	**0.0244**	**0.1182**	**1.37**	0.5115	0.7246	**1.51**	0.5269	0.7455	0.66	0.9329	0.9912	0.79
	GUDCS	0.4083	0.6029	0.71	0.5748	0.7516	0.61	0.1581	0.4886	0.52	0.7747	0.9912	1.59
Bile acid synthesis: primary/alternative pathway	**CA/CDCA**	**0.0058**	**0.0499**	**1.76**	0.5427	0.7381	**1.26**	0.4080	0.6907	1.07	0.7747	0.9912	1.11
Conversion from primary to secondary bile acid by the gut microbiome	DCA/CA	0.6340	0.7942	1.01	0.6761	0.7802	0.95	0.4266	0.6907	0.74	0.8006	0.9912	0.96
	GDCA/CA	0.5180	0.6773	2.16	0.5115	0.7246	4.39	0.6613	0.7495	0.37	0.7490	0.9912	0.35
	TDCA/CA	0.6628	0.7942	2.03	0.4102	0.6766	5.28	0.6150	0.7480	0.39	0.5333	0.9067	0.27
	UDCA/CDCA	0.0838	0.2192	0.65	0.3218	0.6436	0.77	0.3898	0.6907	0.58	0.8796	0.9912	0.78
	LCA/CDCA	0.8529	0.9062	1.13	0.6246	0.7584	1.09	0.7827	0.8064	0.85	0.9329	0.9912	0.77
	GLCA/CDCA	0.1503	0.3408	1.00	0.7114	0.7802	1.09	0.5701	0.7455	1.00	1.0000	1.0000	0.92
	TLCA/CDCA	0.9481	0.9768	1.28	0.6246	0.7584	2.89	0.6380	0.7480	0.54	0.6254	0.9912	0.52
Glycine or taurine conjugation of secondary bile acids by liver enzymes	GDCA/DCA	0.0689	0.1952	1.20	0.1774	0.5026	2.12	0.7828	0.8064	1.39	0.2066	0.5853	0.54
	TDCA/DCA	0.2013	0.3422	1.12	0.1179	0.4009	2.44	0.6380	0.7480	1.49	0.1174	0.4990	0.42
	TUDCA/UDCA	0.4256	0.6029	1.49	0.7472	0.7939	2.32	0.7090	0.7776	0.48	0.5114	0.9067	0.50
	GUDCA/UDCA	0.0674	0.1952	1.70	0.0793	0.3318	2.50	0.5269	0.7455	0.67	0.4094	0.8188	0.55

To calculate *p* values the Mann–Whitney U test was applied. A standard Benjamini-Hochberg method was applied to control the false discovery rate (FDR) for multiple hypothesis testing. FC_1_: the ratio of DN-PD to HC in cohort 1; FC_2_: the ratio of DN-PD to HC in cohort 2; FC_3_: the ratio of DO-PD to DN-PD in cohort 2; FC_4_: the ratio of CO-PD to DN-PD in cohort 2. CA/CDCA was used to evaluate a possible shift in bile acid biosynthesis form primary to the alternative pathway. DCA/CA, GDCA/CA, TDCA/CA, UDCA/CDCA, LCA/CDCA, GLCA/CDCA and TLCA/CDCA were calculated to evaluate the enzymatic active of gut microbiome to covert primary bile acids into secondary bile acids. GDCA/DCA, TDCA/DCA, TUDCA/UDCA and GUDCA/UDCA were used to evaluate the enzymatic activity related to taurine and glycine conjugation of secondary bile acids [22]

### Discriminant model establishment and validation

**Fig. 5:**
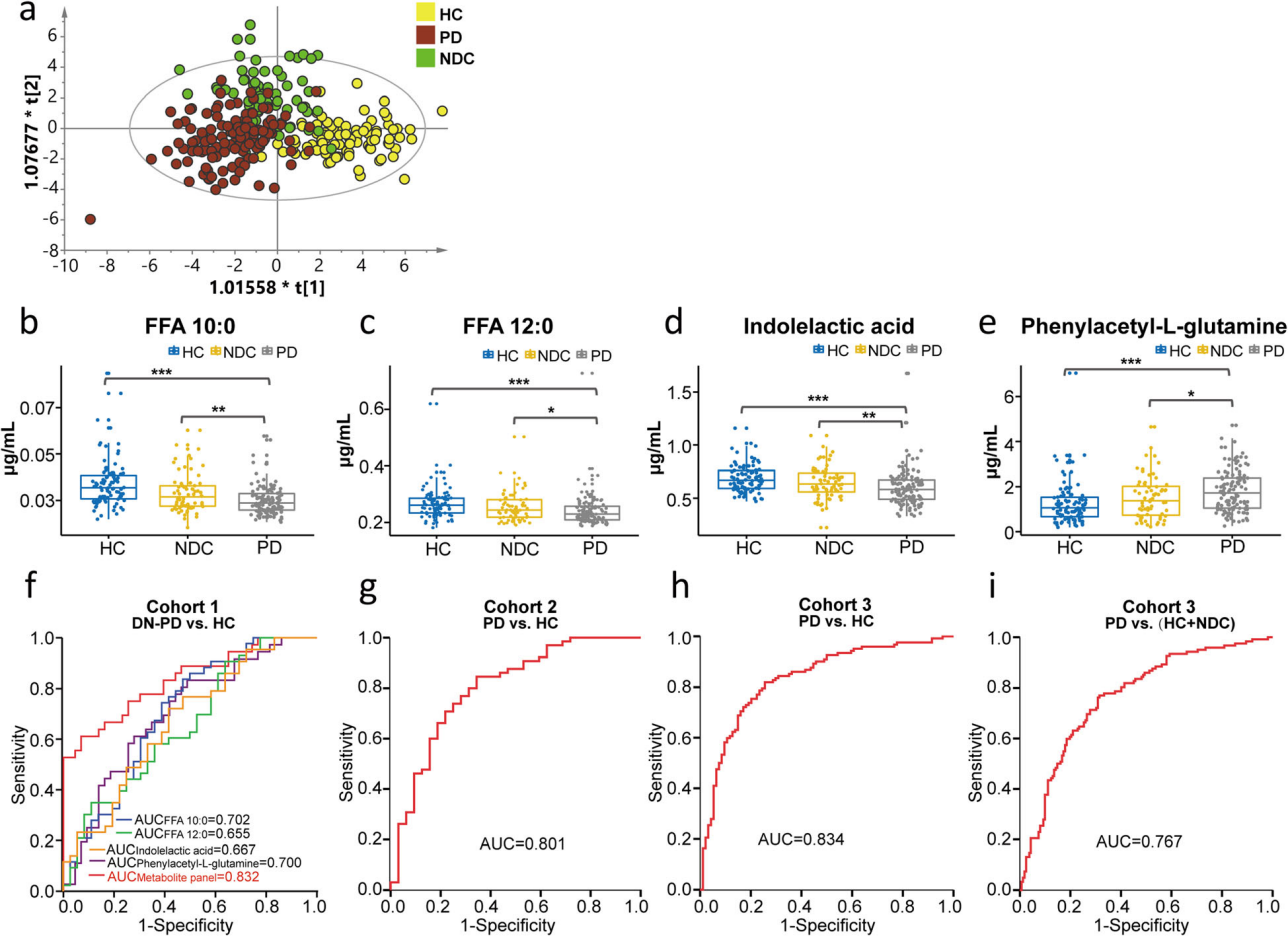
Differential metabolites in PD compared with HC and NDC. **a** OPLS-DA score plot of PD, HC and NDC in cohort 3. R2X = 0.389, R2Y = 0.543, Q2 = 0.347. CV-ANOVA data: *p* = 0, F factor = 17.7056. **b ~ e** Box plots of FFA 10:0, FFA 12:0, indolelactic acid and phenylacetyl-L-glutamine in three groups. Data were expressed as means ± SE. *: *p* < 0.05, **: *p* < 0.01, ***: *p* < 0.001. The ROC curves of the metabolite panel to discriminate PD from control groups. **f** DN-PD vs. HC in cohort 1. The ranges of AUC values at the 95% confidence interval (CI) for FFA 10:0, FFA 12:0, indolelactic acid, phenylacetyl-L-glutamine and metabolite panel were 0.584 ~ 0.820, 0.534 ~ 0.776, 0.547 ~ 0.787, 0.585 ~ 0.816 and 0.742 ~ 0.922, respectively. **g** PD vs. HC in cohort 2. The AUC value ranges from 0.704 to 0.898 at 95% CI. **h** PD vs. HC in cohort 3. The AUC value ranges from 0.778 to 0.889 at 95% CI. **i.** PD vs. (HC + NDC) in cohort 3. The AUC value ranges from 0.711 to 0.822 at 95% CI

To further validate the metabolic changes in PD and identify potential biomarkers, we collected another set of plasma sample for metabolomics analysis. In contrast to the majority of “case-control” studies, we included not only a HC but also a disease control group. Because the disease control group contains a variety of disease types, we applied OPLS-DA to remove the data variation that is independent of categorical variable and decrease the false positive rate. We found that the metabolic profiles of PD, HC and NDC showed a tendency for separation although partially overlapped (Fig. 5a). Six metabolites were identified changed in PD compared to both HC and NDC (**Table S****8**). Besides, we also investigated the impact of antiepileptic medication on the levels of these 6 metabolites. The results demonstrated that there were no significant differences between treated-epilepsy patients and HC (**Table S****9**). Given that the levels of L-3-methoxytyrosine and tyrosine were affected by antiparkinsonian medications, their changes may be induced by antiparkinsonian drugs rather than by PD itself. Thus, they were excluded when developing the discriminant model.

ROC curves of classification models distinguishing PD from HC using the other 4 metabolites (FFA 10:0, FFA 12:0, indolelactic acid and phenylacetyl-glutamine, Fig. 5b-e) were plotted. The AUC values for each metabolite were 0.702, 0.655, 0.667, and 0.700, respectively (Fig. 5f). The discriminant power was improved by combining the 4 metabolites using binary logistic regression analysis with the AUC value reaching to 0.821. Considering the effects of age on metabolite levels, we also included age as a covariate for the development of diagnostic algorithm. The corresponding ROC curve yielded an AUC value of 0.832 when distinguishing DN-PD from HC (Fig. 5f). To validate the effectiveness of the diagnostic model, we also evaluated its discriminating ability in the other two cohorts. As shown in Fig. 5g-h, the ROC curves produced AUC values of 0.801 and 0.834 when distinguishing PD from HC in cohort 2 and cohort 3, respectively. This metabolite panel also showed satisfactory diagnostic performance for distinguishing PD from both HC and NDC with an AUC value of 0.767 (Fig. 5i). Notably, using the same regression equation developed in cohort 1, the metabolic panel also showed a good discriminability across different cohorts (**Figure S****5**). Detailed parameters of the models are given in **Table S****10****-S****13**.

## Discussion

This study presented a comprehensive metabolomic evaluation for PD in 3 independent populations. We investigated the associations of metabolites with gender, age, and stratified PD according to different variables (duration, staging and medication) to better understand the influence of these variables on the biochemical impairment itself. It has been reported that PD patients showed differences between males and females in epidemiological and clinical characteristics, sensitivity to risk factors, and response to treatments [33]. In this study, we found that 24 metabolites including acetylcholine, creatinine, amino acids, PCs and SMs were different between genders in HC but not in DN-PD, which implied that the reported gender-related differences in PD might be associated with different metabolic reprograming in males and females among PD patients. Besides, we developed a discriminant model consisting of FFA 10:0, FFA 12:0, indolelactic acid and phenylacetyl-glutamine to assist to diagnose PD. Although previous studies have reported a few discriminant models derived from urine, CSF, and plasma [18, 19, 34–37], the advantage of this study is that the discriminating power of the developed discriminant model was validated by 3 independent cohorts and the same regression equation, which may have great potential for clinical applications in the future. Our study also highlighted that metabolic disturbances in FFA-related metabolism, biosynthesis of BA and steroid hormone, and amino acid metabolism might be involved in PD pathogenesis.

**Fig. 6:**
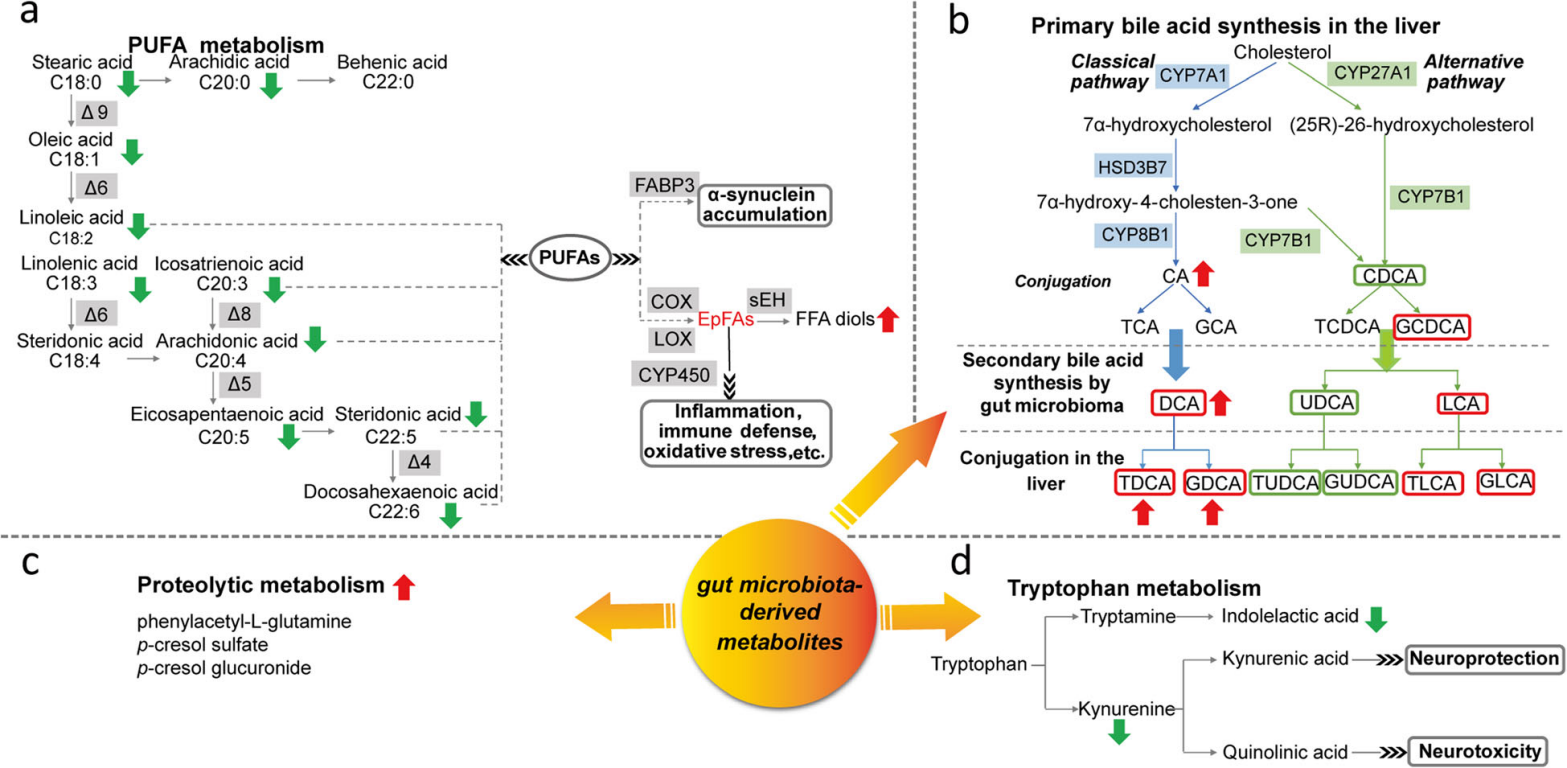
Metabolic disturbances in PD. **a** Alterations in PUFA metabolism. **b.** Alterations in bile acid synthesis pathway. The red frame indicates cytotoxic bile acid, the green frame indicates neuroprotective bile acid. **c** The proteolytic metabolism products were increased in PD. **d** Alterations in tryptophan metabolism

Until recently, alteration of FFA metabolism has been increasingly recognized in PD [5, 38–40]. We found that metabolism of unsaturated FFAs, especially linoleic acid, linolenic acid, and arachidonic acid metabolism, were remarkably perturbed in PD (Fig. 6a). Generally, FFAs are considered to be required for membrane formation, signaling molecule generation and energy supply through beta-oxidation [41]. Recent reports suggested that FFAs, particularly long-chain polyunsaturated FFAs (PUFAs), can bind to monomeric α-synuclein and accelerate the formation of α-synuclein assemblies [42, 43]. It has been reported that fatty acid-binding protein 3 (FABP3) was elevated in the cerebrospinal fluid (CSF) and serum of PD patients and highly expressed in the dopaminergic neurons [44, 45], and was able to promote α-synuclein oligomerization in cultured dopaminergic neurons [42]. Considering this, targeting FABP3 may represent an attractive therapeutic strategy for PD. Prevous study has demonstrated that the developed FABP3 ligands can inhibit arachidonic acid-induced α-synuclein oligomerization in neuro-2A cells [46]. Epoxy fatty acids (EpFAs), the oxidized metabolites of PUFAs, has been found to have potent anti-inflammatory properties [47]. EpFAs can be further metabolized into corresponding diols by soluble epoxide hydrolase (sEH) [48]. It has been reported that overexpression of sEH in the striatum significantly enhanced MPTP-induced neurotoxicity [49]. Diminished PUFAs in PD may be due to the enhancement of their downstream metabolic flux. Therefore, targeting EpFA metabolism may provide novel insight into PD etiology.

To date, a few studies have implicated BAs in PD and mainly focused on the neuroprotection of UDCA and its derivatives [50, 51]. It has been reported that UDCA or TUDCA treatment could improve motor performance, ameliorate mitochondrial dysfunction and neuroinflammation and prevent the decline of striatal dopamine content in various PD models [50–52]. However, knowledge of the potential efficacy of other BAs in PD are still limited. Our study presented a comprehensive analysis of BA profiles in PD patients and identified a range of conjugated and unconjugated BAs, which were significantly disturbed in PD (Fig. 6b). A shift in initial cholesterol metabolism from alternative pathway to primary pathway suggested that altered enzymatic activities lead to excess production of BAs, many of which might be cytotoxic [53]. A previous study has reported reductions of neuroprotective BAs in a prodromal PD mouse model [32]. Together with our findings, it seems that disturbances in BA metabolism might play important roles in the development of PD, even in prodromal stages. Alterations of secondary BAs and BA ratios indicated possible alterations in enzymatic activities of gut microbiota. Notably, the elevations of BAs and BA ratios tend to be alleviated after L-dopa treatment. Increased *Firmicutes* has been found in PD patients [54]. It has been reported that several species of *Firmicutes* in gut can dehydroxylate CA to form DCA, which might be toxic to cells [55]. Determining the specific role and its precise molecular mechanism of each BA in PD might provide novel cues for future therapeutic strategies.

Recently, enteric dysbacteriosis has been recognized as a consistent feature of PD [56]. Abnormalities in the composition and distribution of intestinal bacteria have been suggested in PD patients [57]. It has been postulated that α-synuclein pathology may spread from gut to the brain and contribute to PD etiology, however, the exact mechanism remains unclear [13]. The microbiota-derived metabolites provide a functional readout of the microbiome and can indicate the metabolic interplay among the host, diet, and intestinal bacteria [58]. Apart from BAs, we found a list of microbiota-derived metabolites including proteolytic metabolism products and tryptophan catabolites, which showed significant alterations in PD (Fig. 6c-d). Elevated *p*-cresol sulfate has been detected in the CSF of PD [39, 59]. Recently, Mihai and colleagues demonstrated that *p*-cresol sulfate and phenylacetyl-L-glutamine were increased in the serum of PD and the elevation of these deleterious metabolites was positively correlated with firmer stool and constipation severity among patients [56]. Our study further confirms the increased production of *p*-cresol sulfate and phenylacetyl-L-glutamine in the plasma of PD. Moreover, we identified a novel *p*-cresol metabolite, *p*-cresol glucuronide, a byproduct of protein degradation by gut bacteria, showing significantly increased level in PD. It was revealed that *p*-cresol could inhibit the oxidative respiration and proliferation of colonoscopy cells [56, 60]. Using radio-opaque markers, a study investigated the relationship between colonic transit time and human colonic metabolism and concluded that delayed transit time is accompanied by a shift in colonic metabolism from carbohydrate fermentation to protein catabolism [61]. Therefore, this metabolic alteration may be mechanistically relevant to gastrointestinal disorders in PD patients [62, 63]. A few studies have indicated that the imbalance of kynurenine (Kyn) metabolism, the main catabolic rote of tryptophan, played important roles in PD pathogenesis [19, 64]. Lower levels of KA and KA/Kyn ratio and higher levels of QA and QA/KA ratio were reported in the plasma of PD, indicating a biased Kyn pathway toward producing oxidative stress and excitotoxicity [19]. Apart from Kyn metabolites, we also observed a significantly decreased level of indolelactic acid, another tryptophan catabolite, in PD. Indolelactic acid can be produced by several *Clostridium* species such as *Clostridium sporogenes* and *Clostridium saccharolyticum* [65, 66]. Decreased *Clostridium saccharolyticum* has been reported in fecal samples of PD patients [54]. Besides, alterations of several other *Clostridium* species and *Bacteroides* species that involved in tryptophan catabolism were also observed in PD [54, 67]. A recent study demonstrated that gut microbiota-derived tryptophan catabolites could modulate inflammatory response by attenuating the release of pro-inflammatory cytokines and the cytokine-mediated upregulation of lipogenesis in macrophages and hepatocytes [68]. Previous report also showed anti-neuroinflammatory activities of indole alkaloids in lipopolysaccharide-stimulated BV2 microglial cells [69]. The decreased level of indolelactic acid may contribute to promoting the inflammation in PD.

Both epidemiological and clinical studies have consistently reported that caffeine consumption could reduce the risk of PD [16, 70]. We found significant reduction of its downstream metabolite (trigonelline) in the plasma of PD despite an equivalent caffeine intake to controls. Although there is no statistical significance, we also observed a decreased level of caffeine in PD. Previous studies also reported reduced levels of caffeine and its metabolites in PD and indicated that the reductions have no associations with daily caffeine consumption [3, 71]. A recent work reported that the decrease of these metabolites was also observed in patients with glial cytoplasmic inclusions and neuronal tau accumulation [72]. A common mechanism such as malabsorption from small intestine or abnormal clearance of caffeine may underlie these parkinsonian disorders [72].

Additionally, we documented increased production of cortisol and corticosterone and decreased aldosterone in PD. In the literature, it has been revealed an elevation of cortisol in the plasma and saliva of PD patients [73, 74]. High levels of cortisol can damage substantia nigra striatum system and temporarily aggravate the motor and neuropsychiatric symptoms of PD patients [75, 76]. Corticosterone, the other important glucocorticoid, has been found to impair learning and memory function and cause calcium-induced neurotoxicity in several PD models [77–79]. A recent study indicated that genes related to aldosterone synthesis and secretion were altered in PD [80]. The renin-angiotensin-aldosterone system (RAS) is crucial in the development of hypertension and organ damage, and the activation of brain RAS has been revealed to aggravate the cognitive decline and dopaminergic neuron loss by promoting oxidative stress and inflammation processes [81, 82].

This study provides a comprehensive analysis of metabolic reprogramming in PD. However, it has several limitations. Firstly, PD was diagnosed based on clinical criteria without laboratory confirmation. Further studies to link peripheral metabolic changes to pathophysiology markers, genetic findings and neuroimaging profiles are recommended. Secondly, we only investigated the effects of several commonly used antiparkinsonian treatments, the impacts of other medications cannot be clarified. There are quite few factors such as genetic background, disease history, lifestyle, and diet, etc. which might influence the profiles of the metabolites in PD and controls. To address this issue, future study is necessary to calibrate the levels of metabolites with these factors in a larger cohort investigation.

## Conclusions

In summary, we highlighted that metabolic disturbances in PUFA metabolism, BA and steroid hormone biosynthesis, caffeine metabolism and amino acid metabolism are crucial metabolic events underlying PD. The accumulated microbiota-derived deleterious metabolites including *p*-cresol sulfate, *p*-cresol glucuronide and phenylacetyl-L-glutamine implied an important role of intestinal homeostasis in downstream neurodegenerative processes. These evaluations could improve our understanding of PD etiopathogenesis and facilitate target screening for therapeutic intervention.

## Supplementary Information

**Additional file 1: Table S1.** Concentrations of the stable isotope labeled internal standards in methanol. **Table S2.** Statistical results of FFAs in blank and analytical samples. **Table S3.** Statistical results of differential metabolites between male and female in HC group. **Table S4.** Differential metabolites accountable for the discrimination between drug-naïve PD patients and controls. **Table S5.** Associations between the differential metabolites and disease severity. **Table S6.** Associations between the differential metabolites and duration time. **Table S7.** Associations between the differential metabolites and age. **Table S8.** Statistical results of differential metabolites in PD compared with both HC and NDC groups in cohort 3. **Table S9.** Statistical results of the six selected differential metabolites in treated-epilepsy patients and HC. **Table S10.** Parameters of the binary logistic regression model in cohort 1. **Table S11.** Parameters of the binary logistic regression model in cohort 2. **Table S12.** Parameters of the binary logistic regression model in cohort 3 (PD vs. HC + NDC). **Table S13.** Parameters of the binary logistic regression model in cohort 3 (PD vs. HC). **Figure S1.** Robust assessment of the analytical method across three independent cohorts. **Figure S2.** PCA analysis of the metabolic profiles in male and female of drug-naïve PD and HC. **Figure S3.** Permutation test (999 times) of the PLS-DA models. **Figure S4.** Pathway analysis of the differential metabolites in drug-naïve PD compared with HC. **Figure S5.** The ROC curves of the metabolite panel to discriminate PD from control groups across different cohorts based on the regression equation developed in cohort 1.

## Data Availability

The datasets used and/or analyzed during the current study are available from the corresponding author on reasonable request.

## Abbreviations

QA: Quinolinic acid
KA: Kynurenic acid
BA: Bile acid
HC: Healthy control
NDC: Neurological disease control
IS: Internal standard
QC: Quality control
RSD: Relative standard deviation
PCA: Principal component analysis
PLS-DA: Partial least square discriminant analysis
OPLS-DA: Orthogonal PLS-DA
FDR: False discovery rate
ROC: Receiver operating characteristic
AUC: The area under the curve
DN-PD: Drug-naive PD
PC: Phosphatidylcholine
SM: Sphingomyelin
FFA: Fatty acid
FFAD: FFA amide
DO-PD: L-dopa-treated PD
PR-PD: Pramipexole-treated PD
CO-PD: The combination of L-dopa and pramipexole-treated PD
LPC: Lysophosphatidylcholine
PUFA: Polyunsaturated FFA
FABP3: Fatty acid-binding protein 3
CSF: Cerebrospinal fluid
EpFAs: Epoxy fatty acids
sEH: soluble epoxide hydrolase
RAS: Renin-angiotensin-aldosterone system
Kyn: Kynurenine
LOX: Lipoxygenase
COX: Cyclooxygenases
CA: Cholic acid
CDCA: Chenodeoxycholic acid
DCA: Deoxycholic acid
UDCA: Ursodeoxycholic acid
LCA: Lithocholic acid
GCA: Glycocholic acid
TCA: Taurocholic acid
TCAS: Taurocholic acid 3-sulfate
TCDCA: Taurochenodeoxycholic acid
GCDCA: Glycochenodeoxycholic acid
GCDCS: Glycochenodeoxycholic acid 3-sulfate
TDCA: Taurodeoxycholic acid
GDCA: Glycodeoxycholic acid
TUDCA: Tauroursodeoxycholic acid
GUDCA: Glycoursodeoxycholic acid
GDCS: Glycodeoxycholic acid 3-sulfate
TLCA: Taurolithocholic acid
TLCAS: Taurolithocholic acid 3-sulfate
GLCA: Glycolithocholic acid
TDCS: Taurodeoxycholic acid 3-sulfate
GLCAS: Glycolithocholic acid 3-sulfate
GUDCS: Glycoursodeoxycholic acid 3-sulfate
