# Supplementary material for: Comprehensive metabolic profiling of Parkinson’s disease by liquid chromatography-mass spectrometry
Source: Mol Neurodegener. 2021 Jan 23;16:4. doi: 10.1186/s13024-021-00425-8 (PMC7825156; doi:10.1186/s13024-021-00425-8)
Supplement: Supplementary file 1 — Additional file 1: Table S1. Concentrations of the stable isotope labeled internal standards in methanol. Table S2. Statistical results of FFAs in blank and analytical samples. Table S3. Statistical results of differential metabolites between male and female in HC group. Table S4. Differential metabolites accountable for the discrimination between drug-naïve PD patients and controls. Table S5. Associations between the differential metabolites and disease severity. Table S6. Associations between the differential metabolites and duration time. Table S7. Associations between the differential metabolites and age. Table S8. Statistical results of differential metabolites in PD compared with both HC and NDC groups in cohort 3. Table S9. Statistical results of the six selected differential metabolites in treated-epilepsy patients and HC. Table S10. Parameters of the binary logistic regression model in cohort 1. Table S11. Parameters of the binary logistic regression model in cohort 2. Table S12. Parameters of the binary logistic regression model in cohort 3 (PD vs. HC + NDC). Table S13. Parameters of the binary logistic regression model in cohort 3 (PD vs. HC). Figure S1. Robust assessment of the analytical method across three independent cohorts. Figure S2. PCA analysis of the metabolic profiles in male and female of drug-naïve PD and HC. Figure S3. Permutation test (999 times) of the PLS-DA models. Figure S4. Pathway analysis of the differential metabolites in drug-naïve PD compared with HC. Figure S5. The ROC curves of the metabolite panel to discriminate PD from control groups across different cohorts based on the regression equation developed in cohort 1. [file 13024_2021_425_MOESM1_ESM.docx]

**Comprehensive Metabolic Profiling of Parkinson's Disease by Liquid Chromatography-Mass Spectrometry**

Yaping Shao^1, 2^, Tianbai Li^1, 2^, Zheyi Liu^3^, Xiaolin Wang^3^, Xiaojiao Xu^1, 2^, Song Li^1, 2^, Guowang Xu^3*^ and Weidong Le^1, 2,4*^

^1^ Center for Clinical Research on Neurological Diseases, The First Affiliated Hospital, Dalian Medical University, 193 Lianhe Road, Dalian, China.

^2^ Liaoning Provincial Key Laboratory for Research on the Pathogenic Mechanisms of Neurological Diseases, The First Affiliated Hospital, Dalian Medical University, 193 Lianhe Road, Dalian, China.

^3^ CAS Key Laboratory of Separation Science for Analytical Chemistry, Dalian Institute of Chemical Physics, Chinese Academy of Sciences, 457 Zhongshan Road, 116023, Dalian, China.

^4^ Institute of Neurology, Sichuan Academy of Medical Science-Sichuan Provincial Hospital, Medical School of UESTC, Sichuan, China

**Corresponding Authors:**

* Weidong Le, MD, PhD, Professor and Director, Liaoning Provincial Center for Clinical Research on Neurological Diseases, the First Affiliated Hospital, Dalian Medical University, 193 Lianhe Road, Dalian, China. Tel/Fax: 0086-411-88135850. Email: [wdle_sibs@163.com](mailto:wdle_sibs@163.com)

* Guowang Xu, 457 Zhongshan Road, 116023, Dalian, China. Tel/Fax: 0086-411-84379559. E-mail: [xugw@dicp.ac.cn](mailto:xugw@dicp.ac.cn)

**Chemicals and reagents**

Ultrapure water was from Milli-Q water purification system (Millipore, Billerica, MA, USA). Methanol and acetonitrile were HPLC-grade and obtained from Merck (Darmstodt, Germany). Mobile phase additive formic acid was purchased from Fluka (Germany). Internal standards and ammonium bicarbonate were products of Sigma-Aldrich (St. Louis, MO, USA).

**Chromatographic conditions for metabolomics study**

Chromatographic separation of the extracts was performed on an Ultra Performance Liquid Chromatography (UPLC, Waters Corporation, Manchester, UK) system. Both ESI+ mode and ESI- mode were used to facilitate the ionization and improve the detection sensitivity of alkaline compounds and acidic compounds, respectively. In ESI+ mode, extracts were retained and gradient eluted by an ACQUITY UPLC BEH C8 column, and the mobile phases were water (A) and acetonitrile (B) containing 0.1% formic acid solution. In ESI- mode analysis, extracts were retained and gradient eluted from ACQUITY UPLC HSS T3 column and the mobile phases were water (C) and 95% methanol (D) containing 6.5 mM ammonium bicarbonate. For metabolomics analysis in cohort 1 and cohort 2, conventional elution gradient was employed^1^. In ESI+ mode, the elution gradient initiated with 5% B for 1.0 min, linearly increased to 100% B at 24 min and maintained 4 min, then went back to 5% B in 0.1 min for post equilibration. In ESI- mode, the elution gradient started with 2% D for 1.0 min, and increased to 100% D at 18 min and maintained for 4 min, then decreased to 2% D in 0.1 min. The delivery flow rate was 0.35 mL/min, and the column temperature was 50℃. For metabolomics analysis in cohort 3, a fast elution gradient was used^2^. In ESI+ mode, the elution gradient was started with 5% B for 0.5 min, and linearly increased to 40 % B at 2 min, to 100% B at 8 min and maintained for 2 min, then went back to 5 % B in 0.1 min and held for 1.9 min. In ESI- modes, the elution gradient started with 2% D and maintained for 0.5min, then linearly increased to 40% D at 2min, to 100%D at 8min and held for 2min, then decreased to 2% D in 0.1min and maintained for 1.9 min for post equilibration. The delivery flow rate was 0.40 mL/min. The column temperature was 60℃.

**MS conditions for metabolomics study**

The MS conditions were as follows: gas 1, gas 2 and curtain gas were set at 50 psi, 50 psi and 35 psi, respectively. Ion source temperature was 500℃, and ion spray voltage floating was 5,500 V in ESI+ mode and -4,500 V in ESI- mode.

**Bile acid targeted analysis**

Because information of low abundance of bile acid may be lost during the process of automatic peak alignment, we performed targeted extraction and integration of the chromatographic peak of bile acids in plasma samples. Briefly, data matrix including exact mass and secondary MS/MS information of metabolites in a QC sample operated in high, medium, and low collision energy were exported to .mgf files using Peakview software (version 1.2.0.3, Applied Biosystems). Twenty-two bile acids were annotated using an in-house database by comparison of the ion features in QC samples with the reference chemical standards using OSI/SMMS software. A qualitative table including retention time, exact mass and catalogue of bile acid was generated and imported into Peakview software to extract the chromatographic peak of each bile acid in the analytical samples. MultiQuant (version 2.1, Applied Biosystems) software was used to construct quantitative method, and view and integrate chromatographic peak of each bile acid. The integration parameters: noise percentage, 40.0%, baseline sub. window, 2.00 min, peak splitting, 1 point. A peak table including peak area, sample name and catalogue of bile acid was generated and used for the statistical analysis.

**Table S1. Concentrations of the stable isotope labeled internal standards in methanol.**

| **Internal standards** | **Concentration (μg/mL)** |
| --- | --- |
| Carnitine C2:0-d_3_ | 0.10 |
| Carnitine C10:0-d_3_ | 0.10 |
| Carnitine C16:0-d_3_ | 0.15 |
| LPC 12:0 | 0.75 |
| FFA C16:0-d_3_ | 2.50 |
| FFA C18:0-d_3_ | 2.50 |
| CDCA-d_4_ | 0.80 |
| CA-d_4_ | 0.50 |
| Tryptophan-d_5_ | 4.00 |
| Phenylalanine-d_5_ | 3.00 |
| Leucine-d_3_ | 4.00 |
| Choline-d_4_ | 1.00 |
| Tridecanoic acid | 2.00 ~ 5.00 |
| Succinic acid-d_4_ | 2.00 ~ 5.00 |

**Table S2. Statistical results of FFAs in blank and analytical samples.**

| **Name** | **Intensity in blank sample** | **Intensity in QC sample** | **B/Q (%)** | **RSD-IS (%)** |
| --- | --- | --- | --- | --- |
| FFA 9:0 | 7.21E+02 | 4.69E+04 | 1.54 | 12.30 |
| FFA 10:0 | 2.10E+03 | 2.78E+04 | 7.56 | 7.50 |
| FFA 11:1 | NA | 1.27E+04 | NA | 9.05 |
| FFA 12:0 | 6.45E+04 | 2.80E+05 | 23.03 | 9.32 |
| FFA 14:0 | 8.73E+04 | 3.67E+05 | 23.76 | 5.43 |
| FFA 14:1 | NA | 6.65E+04 | NA | 3.18 |
| FFA 15:0 | 2.42E+04 | 6.98E+04 | 34.73 | 6.34 |
| FFA 16:0 | 8.42E+05 | 6.38E+06 | 13.19 | 3.50 |
| FFA 16:1 | 9.55E+03 | 9.03E+05 | 1.06 | 2.18 |
| FFA 16:2 | 1.13E+02 | 3.70E+04 | 0.31 | 4.38 |
| FFA 17:0 | 1.74E+04 | 1.50E+05 | 11.60 | 4.05 |
| FFA 17:1 | 2.05E+03 | 6.51E+04 | 3.16 | 4.15 |
| FFA 18:0 | 3.36E+05 | 3.35E+06 | 10.04 | 3.11 |
| FFA 18:1 | 2.49E+05 | 9.50E+06 | 2.62 | 1.93 |
| FFA 18:2 | 4.00E+04 | 7.77E+06 | 0.52 | 2.49 |
| FFA 18:3 | 1.09E+03 | 7.97E+05 | 0.14 | 3.17 |
| FFA 19:1 | 5.43E+02 | 3.47E+04 | 1.57 | 6.06 |
| FFA 20:0 | 9.47E+03 | 7.83E+04 | 12.10 | 3.01 |
| FFA 20:1 | 2.74E+03 | 2.37E+05 | 1.15 | 2.45 |
| FFA 20:2 | 1.62E+02 | 2.57E+05 | 0.06 | 5.21 |
| FFA 20:3 | 8.65E+01 | 1.36E+05 | 0.06 | 4.76 |
| FFA 20:4 | NA | 2.98E+05 | NA | 3.18 |
| FFA 20:5 | 2.29E+01 | 6.19E+04 | 0.04 | 4.17 |
| FFA 22:0 | 4.57E+03 | 1.05E+05 | 4.36 | 5.08 |
| FFA 22:1 | 4.02E+03 | 5.72E+04 | 7.03 | 24.32 |
| FFA 22:2 | 7.66E+02 | 2.52E+04 | 3.04 | 8.69 |
| FFA 22:4 | 1.06E+07 | 1.40E+07 | 75.34 | 15.82 |
| FFA 22:5 | 1.91E+03 | 2.82E+05 | 0.68 | 4.71 |
| FFA 22:6 | NA | 7.84E+05 | NA | 2.58 |
| FFA 24:0 | 9.11E+03 | 1.41E+05 | 6.47 | 4.27 |
| FFA 24:1 | NA | 1.06E+05 | NA | 4.34 |
| FFA 24:2 | 7.57E+01 | 2.73E+04 | 0.28 | 4.86 |
| FFA 24:4 | NA | 7.93E+04 | NA | 11.09 |
| FFA 24:5 | NA | 2.53E+04 | NA | 7.83 |
| FFA 24:6 | NA | 1.43E+04 | NA | 6.99 |

NA: Not available, which indicates that the FFA cannot be detected in blank samples. Intensity in blank sample: the average of 3 blank samples. Intensity in QC sample: the average of 28 QC samples. B/Q: the ratio of the intensity of FFA in blank sample to that in QC samples. RSD-IS: the RSD of FFA in 28 QC samples after IS calibration.

**Table S3. Statistical results of differential metabolites between male and female in HC group.**

| **Metabolite** | **HC** | | | **drug-naïve PD** | | |
| --- | --- | --- | --- | --- | --- | --- |
|  | ***p*** | **FDR** | **m/f** | ***p*** | **FDR** | **m/f** |
| 3-(1-Pyrazolyl)-L-alanine | 0.0002 | 0.0393 | 1.20 | 0.8113 | 0.9550 | 0.97 |
| acetylcholine | 0.0017 | 0.0595 | 1.28 | 0.4169 | 0.8889 | 0.94 |
| bilirubin | 0.0087 | 0.1040 | 1.39 | 0.1973 | 0.7252 | 1.30 |
| creatinine | 0.0018 | 0.0595 | 1.18 | 0.0432 | 0.5049 | 1.12 |
| arginine | 0.0065 | 0.0921 | 1.36 | 0.9619 | 0.9917 | 0.99 |
| leucine | 0.0045 | 0.0840 | 1.15 | 0.6907 | 0.9407 | 0.99 |
| methionine | 0.0012 | 0.0595 | 1.25 | 0.7623 | 0.9407 | 1.01 |
| valine | 0.0038 | 0.0785 | 1.10 | 0.7867 | 0.9407 | 1.02 |
| phenylalanine | 0.0008 | 0.0595 | 1.17 | 0.7382 | 0.9407 | 1.01 |
| N-acetyl-glutamine | 0.0017 | 0.0595 | 1.09 | 0.5347 | 0.9104 | 1.02 |
| γ-glu-leu | 0.0116 | 0.1142 | 1.14 | 0.7867 | 0.9407 | 1.07 |
| 5a-dihydrotestosterone sulfate | 0.0164 | 0.1481 | 1.57 | 0.1973 | 0.7252 | 1.31 |
| carnitine C18:3 | 0.0056 | 0.0846 | 1.39 | 0.3989 | 0.8889 | 1.23 |
| LPC 18:2 | 0.0116 | 0.1142 | 1.18 | 0.2584 | 0.7684 | 1.12 |
| LPC 20:2 | 0.0094 | 0.1062 | 1.23 | 0.1475 | 0.6952 | 1.18 |
| phosphate | 0.0033 | 0.0785 | 0.88 | 0.8113 | 0.9550 | 1.02 |
| 1,3-dimethyluric acid | 0.0101 | 0.1086 | 0.67 | 0.7867 | 0.9407 | 0.98 |
| PC 32:2 | 0.0070 | 0.0934 | 0.79 | 0.0772 | 0.5456 | 0.90 |
| PC 33:1 | 0.0052 | 0.0840 | 0.77 | 0.5347 | 0.9104 | 1.01 |
| PC 33:2 | 0.0052 | 0.0840 | 0.77 | 0.5140 | 0.9104 | 1.02 |
| SM 32:2 | 0.0009 | 0.0595 | 0.73 | 0.0079 | 0.2933 | 0.77 |
| SM 34:2 | 0.0076 | 0.0949 | 0.84 | 0.4937 | 0.9104 | 0.92 |
| SM 35:2 | 0.0038 | 0.0785 | 0.81 | 0.0773 | 0.5456 | 0.86 |
| SM 36:3 | 0.0153 | 0.1442 | 0.87 | 0.9113 | 0.9917 | 1.00 |

To calculate *p* values the Mann–Whitney U test was adopted. A standard Benjamini-Hochberg method was applied to control the false discovery rate (FDR) for multiple hypothesis testing. Metabolites with a *p* value < 0.05 and FDR < 0.15 were considered to be statistically significantly changed. PC: phosphatidylcholine; SM: sphingomyelin. LPC: lysophosphatidylcholine. γ-glu-leu: gamma-glutamyl-leucine.

**Table S4. Differential metabolites accountable for the discrimination between drug-naïve PD patients and controls.**

| **Pathway** | **Metabolite** | ***m/z*** | **t_R_/min** | **Detection mode** | ***p* value** | **FDR** | **VIP value** | **Fold change** |
| --- | --- | --- | --- | --- | --- | --- | --- | --- |
| **Amino acid metabolism** |  |  |  |  |  |  |  |  |
|  | Phenylacetyl-L-glutamine | 265.1172 | 3.56 | ESI+ | 0.0023 | 0.0209 | 1.65 | 1.57 |
| Tryptophan metabolism | Kynurenine | 209.0914 | 1.36 | ESI+ | 0.0113 | 0.0620 | 1.71 | 0.86 |
|  | Indolelactic acid | 204.0671 | 3.97 | ESI- | 0.0109 | 0.0618 | 1.64 | 0.81 |
| Methionine metabolism | N-Acetyl-L-methionine | 192.0639 | 1.36 | ESI+ | 0.0035 | 0.0284 | 1.95 | 0.85 |
| Proline metabolism | Proline | 116.0708 | 0.73 | ESI+ | 0.0401 | 0.1395 | 1.26 | 1.14 |
| Tyrosine biotransformation | *p*-Cresol glucuronide | 283.0838 | 4.29 | ESI- | 0.0021 | 0.0204 | 1.78 | 2.15 |
|  | *p*-Cresol sulfate | 187.0079 | 5.28 | ESI+ | 0.0278 | 0.1065 | 1.12 | 1.08 |
| **FFA metabolism** |  |  |  |  |  |  |  |  |
|  | FFA 11:1 | 183.1399 | 12.41 | ESI- | 0.0061 | 0.0461 | 1.37 | 0.76 |
|  | FFA 19:1 | 295.2638 | 17.48 | ESI- | 0.0179 | 0.0861 | 1.25 | 0.78 |
|  | FFA 14:1 | 225.1846 | 15.11 | ESI- | 0.0100 | 0.0614 | 1.21 | 0.77 |
| FFA biosynthesis | FFA 10:0 | 171.1402 | 12.37 | ESI- | 0.0021 | 0.0204 | 1.38 | 0.81 |
|  | FFA 12:0 | 199.1709 | 14.26 | ESI- | 0.0184 | 0.0866 | 1.52 | 0.91 |
|  | FFA 18:0 | 283.2625 | 17.64 | ESI- | 0.0012 | 0.0164 | 1.80 | 0.87 |
| Biosynthesis of unsaturated FFA | FFA 16:2 | 251.2007 | 15.48 | ESI- | 0.0000 | 0.0044 | 2.37 | 0.59 |
|  | FFA 22:5 | 329.2469 | 16.93 | ESI- | 0.0002 | 0.0058 | 1.94 | 0.63 |
| Linoleic acid metabolism | FFA 18:1 | 281.2482 | 17.05 | ESI- | 0.0199 | 0.0882 | 1.24 | 0.80 |
|  | FFA 18:2 | 279.2333 | 16.50 | ESI- | 0.0106 | 0.0616 | 1.62 | 0.73 |
|  | FFA 18:3 | 277.2179 | 16.00 | ESI- | 0.0331 | 0.1187 | 1.06 | 0.77 |
|  | FFA 20:5 | 301.2165 | 16.14 | ESI- | 0.0014 | 0.0164 | 2.10 | 0.61 |
|  | FFA 20:2 | 307.2605 | 17.44 | ESI- | 0.0090 | 0.0579 | 1.16 | 0.76 |
|  | FFA 22:6 | 327.2326 | 16.66 | ESI- | 0.0000 | 0.0040 | 2.28 | 0.60 |

(Table S4 continued)

| Arachidonic acid metabolism | FFA 20:0 | 311.2958 | 18.34 | ESI- | 0.0307 | 0.1120 | 1.23 | 0.87 |
| --- | --- | --- | --- | --- | --- | --- | --- | --- |
|  | FFA 20:3 | 305.2485 | 16.94 | ESI- | 0.0000 | 0.0045 | 2.05 | 0.63 |
|  | FFA 20:4 | 303.2329 | 16.54 | ESI- | 0.0004 | 0.0081 | 1.91 | 0.65 |
| Beta oxidation of FFAs | carnitine 10:1 | 314.2321 | 9.15 | ESI+ | 0.0078 | 0.0515 | 1.12 | 0.71 |
|  | carnitine 12:0 | 344.2783 | 11.68 | ESI+ | 0.0022 | 0.0204 | 1.03 | 0.78 |
|  | carnitine 12:1 | 342.2637 | 10.97 | ESI+ | 0.0014 | 0.0164 | 1.23 | 0.75 |
|  | carnitine 14:2 | 368.2789 | 11.80 | ESI+ | 0.0034 | 0.0284 | 1.18 | 0.74 |
|  | carnitine 14:3 | 366.2625 | 10.88 | ESI+ | 0.0095 | 0.0596 | 1.38 | 0.74 |
| **Fatty acid amide metabolism** |  |  |  |  |  |  |  |  |
|  | FFAD 20:0 | 312.3256 | 19.64 | ESI+ | 0.0008 | 0.0157 | 1.96 | 0.80 |
|  | FFAD 20:1 | 310.3100 | 18.64 | ESI+ | 0.0000 | 0.004 | 2.09 | 0.62 |
|  | FFAD 22:0 | 340.3574 | 20.88 | ESI+ | 0.0037 | 0.0292 | 1.64 | 0.84 |
|  | FFAD 22:1 | 338.3412 | 19.92 | ESI+ | 0.0014 | 0.0164 | 2.07 | 0.84 |
| **Lipids metabolism** |  |  |  |  |  |  |  |  |
| Glycerophospholipid metabolism | PC 32:2 | 730.5384 | 21.37 | ESI+ | 0.0005 | 0.0111 | 2.17 | 0.82 |
|  | PC 32:3 | 728.5233 | 20.26 | ESI+ | 0.0194 | 0.0877 | 1.53 | 0.85 |
|  | PC 34:2 | 758.5706 | 22.22 | ESI+ | 0.0069 | 0.0502 | 1.64 | 0.94 |
|  | PC 34:3 | 756.5561 | 21.60 | ESI+ | 0.0011 | 0.0164 | 2.00 | 0.82 |
|  | PC 34:4 | 754.5350 | 21.35 | ESI+ | 0.0221 | 0.0942 | 1.60 | 0.81 |
| Sphingolipid metabolism | SM 32:2 | 673.5267 | 19.16 | ESI+ | 0.0122 | 0.0629 | 1.56 | 0.83 |
| **Steroid hormone biosynthesis** |  |  |  |  |  |  |  |  |
|  | Cortisol | 363.2162 | 7.64 | ESI+ | 0.0165 | 0.0830 | 1.71 | 1.21 |
|  | Aldosterone | 361.2006 | 7.67 | ESI+ | 0.0271 | 0.1065 | 1.37 | 0.92 |
|  | Corticosterone | 347.2208 | 8.82 | ESI+ | 0.0002 | 0.0058 | 2.02 | 1.83 |

(Table S4 continued)

| **Others** |  |  |  |  |  |  |  |  |
| --- | --- | --- | --- | --- | --- | --- | --- | --- |
| Caffeine metabolism | Trigonelline | 138.0548 | 0.75 | ESI+ | 0.0119 | 0.0625 | 1.28 | 0.53 |
| Ubiquinone Biosynthesis | Ubiquinone 1 | 251.1263 | 11.34 | ESI+ | 0.0030 | 0.0261 | 1.97 | 0.73 |
| Pantothenate and CoA biosynthesis | Pantothenic acid | 220.1160 | 1.71 | ESI+ | 0.0174 | 0.0857 | 1.32 | 0.85 |
| Citrate cycle (TCA cycle) | cis-Aconitic acid | 173.0096 | 0.56 | ESI- | 0.0078 | 0.0515 | 1.64 | 0.83 |
| Pyrimidine metabolism | Uridine | 243.0632 | 1.04 | ESI- | 0.0300 | 0.1120 | 1.23 | 0.91 |
| Redox metabolism | Biliverdin | 583.2545 | 10.49 | ESI+ | 0.0003 | 0.0081 | 1.99 | 0.70 |
| Multiple biological pathways, like alanine metabolism, etc. | Phosphate | 98.9834 | 0.82 | ESI+ | 0.0012 | 0.0164 | 2.00 | 1.10 |
| Inositol Metabolism | Myoinositol | 179.0569 | 0.74 | ESI- | 0.0264 | 0.1065 | 1.16 | 0.76 |

To calculate *p* values the Mann–Whitney U test was adopted. A standard Benjamini-Hochberg method was applied to control the false discovery rate (FDR) for multiple hypothesis testing. Fold change represents the ratio of the mean of each metabolite in PD to the average of HC. FFA: fatty acid; FFAD: fatty acid amide; PC: phosphatidylcholine; SM: sphingomyelin.

**Table S5. Associations between the differential metabolites and disease severity.**

R2: 0.158

F: 6.357

Significance = 0.017

|  | **B** | **SE** | **t** | **Significance** | **Tolerance** | **VIF** |
| --- | --- | --- | --- | --- | --- | --- |
| constant | 1.547 | 0.222 | 6.965 | 0.000 |  |  |
| FFA 14:1 | 7.985 | 3.167 | 2.521 | 0.017 | 1.000 | 1.000 |

Multiple linear regression was applied. R2: coefficient of determination, which is used to determine the fitness of linear equation. Significance (*p* value) is used to test whether the linear regression equation is statistically significant. B: partial regression coefficient, SE: standard error. Tolerance and VIF (variance inflation factor) were collinearity statistics used to determine whether collinearity exists between independent variables. FFA: fatty acid.

**Table S6. Associations between the differential metabolites and duration time.**

R2: 0.692

F: 13.476

Significance = 0.000

|  | **B** | **SE** | **t** | **Significance** | **Tolerance** | **VIF** |
| --- | --- | --- | --- | --- | --- | --- |
| constant | -11.676 | 4.995 | -2.338 | 0.026 |  |  |
| FFA 20:5 | -35.628 | 14.361 | -2.481 | 0.019 | 0.573 | 1.744 |
| FFA 14:1 | 99.481 | 16.363 | 6.080 | 0.000 | 0.624 | 1.602 |
| PC 34:2 | 0.661 | 0.255 | 2.590 | 0.015 | 0.894 | 1.118 |
| indolelactic acid | 19.463 | 7.460 | 2.609 | 0.014 | 0.769 | 1.301 |
| FFA 16:2 | -124.828 | 49.107 | -2.542 | 0.016 | 0.501 | 1.996 |

Multiple linear regression was applied. R2: coefficient of determination, which is used to determine the fitness of linear equation. Significance (*p* value) is used to test whether the linear regression equation is statistically significant. B: partial regression coefficient, SE: standard error. Tolerance and VIF (variance inflation factor) were collinearity statistics used to determine whether collinearity exists between independent variables. FFA: fatty acid. The levels of FFA 14:1, PC 34:2 and indolelactic acid were positively associated with duration time of the disease, whereas the levels of FFA 20:5 and FFA 16:2 showed negative associations.

**Table S7. Associations between the differential metabolites and age.**

|  |  | **B** | **SE** | **t** | **Significance** | **Tolerance** | **VIF** |
| --- | --- | --- | --- | --- | --- | --- | --- |
| **HC** | constant | 65.316 | 6.850 | 9.535 | 0.000 |  |  |
|  | aldosterone | -340.334 | 90.206 | -3.773 | 0.001 | 0.886 | 1.129 |
|  | pantothenic acid | 76.961 | 20.766 | 3.706 | 0.001 | 0.944 | 1.059 |
|  | N-acetyl-L-methionine | 137.303 | 58.750 | 2.337 | 0.025 | 0.892 | 1.121 |
| **drug-naïve PD** | constant | 93.355 | 9.054 | 10.311 | 0.000 |  |  |
|  | FFA 12:0 | -96.517 | 29.842 | -3.234 | 0.003 | 1.000 | 1.000 |

Multiple linear regression was applied. R2: coefficient of determination, which is used to determine the fitness of linear equation. The R2 values were 0.573 and 0.235 for the models in HC and drug-naïve PD. Significance (*p* value) is used to test whether the linear regression equation is statistically significant. Significances were 0.000 and 0.003 for the models in HC and drug-naïve PD. F factors were 17.430 and 10.460 for the two models. B: partial regression coefficient, SE: standard error. Tolerance and VIF (variance inflation factor) were collinearity statistics used to determine whether collinearity exists between independent variables. FFA: fatty acid.

**Table S8. Statistical results of differential metabolites in PD compared with both HC and NDC groups in cohort 3.**

|  | **PD vs. HC** | | | | **PD vs. NDC** | | | | **NDC vs. HC** | | | |
| --- | --- | --- | --- | --- | --- | --- | --- | --- | --- | --- | --- | --- |
|  | ***p* value** | **FDR** | **VIP** | **FC_1_** | ***p* value** | **FDR** | **VIP** | **FC_2_** | ***p* value** | **FDR** | **VIP** | **FC_3_** |
| L-3-methoxytyrosine | <0.0001 | <0.0001 | 1.98 | 14.68 | <0.0001 | <0.0001 | 3.29 | 19.90 | 0.5836 | 0.7032 | 0.66 | 0.74 |
| Tyrosine | <0.0001 | 0.0004 | 1.52 | 1.17 | <0.0001 | 0.0052 | 2.75 | 1.19 | 0.7847 | 0.8493 | 0.33 | 0.99 |
| Indolelactic acid | <0.0001 | <0.0001 | 1.52 | 0.87 | 0.0063 | 0.1227 | 1.41 | 0.92 | 0.0844 | 0.1851 | 0.95 | 0.94 |
| Phenylacetyl-L-glutamine | <0.0001 | <0.0001 | 1.46 | 1.41 | 0.0187 | 0.1533 | 1.53 | 1.20 | 0.0622 | 0.1493 | 0.78 | 1.17 |
| FFA 10:0 | <0.0001 | <0.0001 | 2.20 | 0.80 | 0.0078 | 0.1227 | 2.03 | 0.91 | 0.0012 | 0.0095 | 1.47 | 0.89 |
| FFA 12:0 | <0.0001 | <0.0001 | 1.13 | 0.91 | 0.0386 | 0.2318 | 1.03 | 0.95 | 0.0537 | 0.1320 | 0.74 | 0.95 |

To calculate *p* values the Mann–Whitney U test was adopted. A standard Benjamini-Hochberg method was applied to control the false discovery rate (FDR) for multiple hypothesis testing. FC1, the ratio of PD to HC; FC2, the ratio of PD to NDC; FC3, the ratio of NDC to HC. The metabolites including L-3-metoxytyrosine, tyrosine, indolelactic acid, phenylacetyl-L-glutamine and FFA 12:0 were changed in PD compared to HC and NDC, but had no significant differences between HC and NDC. The metabolite FFA 10:0 showed a gradual decrease from HC, NDC to PD.

**Table S9. Statistical results of the six selected differential metabolites in** **treated-epilepsy patients and HC.**

|  | ***p* value** | **FDR** | **Fold changed** |
| --- | --- | --- | --- |
| L-3-Methoxytyrosine | 0.2647 | 0.4390 | 0.72 |
| Tyrosine | 0.8564 | 0.8959 | 0.99 |
| Indolelactic acid | 0.8747 | 0.9104 | 0.95 |
| Phenylacetyl-L-glutamine | 0.4513 | 0.6220 | 1.31 |
| FFA 10:0 | 0.2313 | 0.3999 | 0.88 |
| FFA 12:0 | 0.9953 | 0.9953 | 0.98 |

To calculate *p* values the Mann–Whitney U test was adopted. A standard Benjamini-Hochberg method was applied to control the false discovery rate (FDR) for multiple hypothesis testing. Fold change indicated the average of metabolites levels in treated-epilepsy patients to that of the HC. All the six metabolites had no significant difference between treated-epilepsy and HC, suggested that the epilepsy-specific treatment can not affect the levels of these six metabolites.

**Table S10. Parameters of the binary logistic regression model in cohort 1.**

chi-square: *p* = 2.0511E-05

Cox & Snell R2:0.3096

Nagelkerke R2:0.4139

Hosmer & Lemeshow test: *p* = 0.6232

Probability: 0.7215

|  | **B** | **SE** | **Wals** | **df** | **Sig.** | **Exp(B)** |
| --- | --- | --- | --- | --- | --- | --- |
| age | -0.056 | 0.038 | 2.155 | 1 | 0.1421 | 0.9460 |
| FFA 10:0 | -37.552 | 32.302 | 1.351 | 1 | 0.2450 | 4.9156E-17 |
| FFA12:0 | -11.893 | 6.480 | 3.369 | 1 | 0.0664 | 6.8361E-06 |
| indolelactic acid | -9.310 | 3.427 | 7.382 | 1 | 0.0066 | 9.0499E-05 |
| phenylacetyl-glutamine | 1.530 | 0.490 | 9.738 | 1 | 0.0018 | 4.6167 |
| constant | 9.349 | 3.488 | 7.186 | 1 | 0.0073 | 11492.2706 |

B: partial regression coefficient; SE: standard error; Wals: Wald statistic; Exp(B): odds ratios.

**Table S11. Parameters of the binary logistic regression model in cohort 2.**

chi-square: *p* = 1.7892E-05

Cox & Snell R2:0.2628

Nagelkerke R2:0.3657

Hosmer & Lemeshow test: *p* = 0.7667

Probability: 0.7526

|  | **B** | **SE** | **Wals** | **df** | **Sig.** | **Exp(B)** |
| --- | --- | --- | --- | --- | --- | --- |
| age | 0.022 | 0.029 | 0.571 | 1 | 0.4497 | 1.0223 |
| FFA 10:0 | -21.626 | 28.284 | 0.585 | 1 | 0.4445 | 4.0542E-10 |
| FFA12:0 | -33.143 | 11.201 | 8.756 | 1 | 0.0031 | 4.0373E-15 |
| indolelactic acid | -2.723 | 1.535 | 3.145 | 1 | 0.0761 | 0.0657 |
| phenylacetyl-glutamine | 0.670 | 0.397 | 2.841 | 1 | 0.0919 | 1.9534 |
| constant | 4.061 | 2.077 | 3.823 | 1 | 0.0506 | 58.0270 |

B: partial regression coefficient; SE: standard error; Wals: Wald statistic; Exp(B): odds ratios.

**Table S12. Parameters of the binary logistic regression model in cohort 3 (PD vs. HC+NDC).**

chi-square: *p* = 1.7316E-11

Cox & Snell R2:0.1883

Nagelkerke R2:0.2528

Hosmer & Lemeshow test: *p* = 0.5265

Probability: 0.7183

|  | **B** | **SE** | **Wals** | **df** | **Sig.** | **Exp(B)** |
| --- | --- | --- | --- | --- | --- | --- |
| age | -0.024 | 0.016 | 2.149 | 1 | 0.1427 | 0.9767 |
| FFA 10:0 | -101.312 | 26.306 | 14.833 | 1 | 0.0001 | 1.0013E-44 |
| FFA12:0 | 0.975 | 3.143 | 0.096 | 1 | 0.7563 | 2.6521 |
| indolelactic acid | -2.986 | 0.977 | 9.344 | 1 | 0.0022 | 0.0505 |
| phenylacetyl-glutamine | 0.483 | 0.166 | 8.493 | 1 | 0.0036 | 1.6216 |
| constant | 5.483 | 1.402 | 15.307 | 1 | 9.1400E-05 | 240.6240 |

B: partial regression coefficient; SE: standard error; Wals: Wald statistic; Exp(B): odds ratios.

**Table S13. Parameters of the binary logistic regression model in cohort 3 (PD vs. HC).**

chi-square: *p* = 3.3877E-14

Cox & Snell R2:0.2845

Nagelkerke R2:0.3815

Hosmer & Lemeshow test: *p* = 0.4100

Probability: 0.7685

|  | **B** | **SE** | **Wals** | **df** | **Sig.** | **Exp(B)** |
| --- | --- | --- | --- | --- | --- | --- |
| age | -0.038 | 0.020 | 3.532 | 1 | 0.0602 | 0.9628 |
| FFA 10:0 | -140.714 | 32.532 | 18.709 | 1 | 1.5224E-05 | 7.7408E-62 |
| FFA12:0 | 1.220 | 3.484 | 0.123 | 1 | 0.7262 | 3.3871 |
| indolelactic acid | -3.785 | 1.249 | 9.180 | 1 | 0.0024 | 0.0227 |
| phenylacetyl-glutamine | 0.638 | 0.233 | 7.512 | 1 | 0.0061 | 1.8924 |
| constant | 8.631 | 1.843 | 21.927 | 1 | 2.8320E-06 | 5603.0387 |

B: partial regression coefficient; SE: standard error; Wals: Wald statistic; Exp(B): odds ratios.


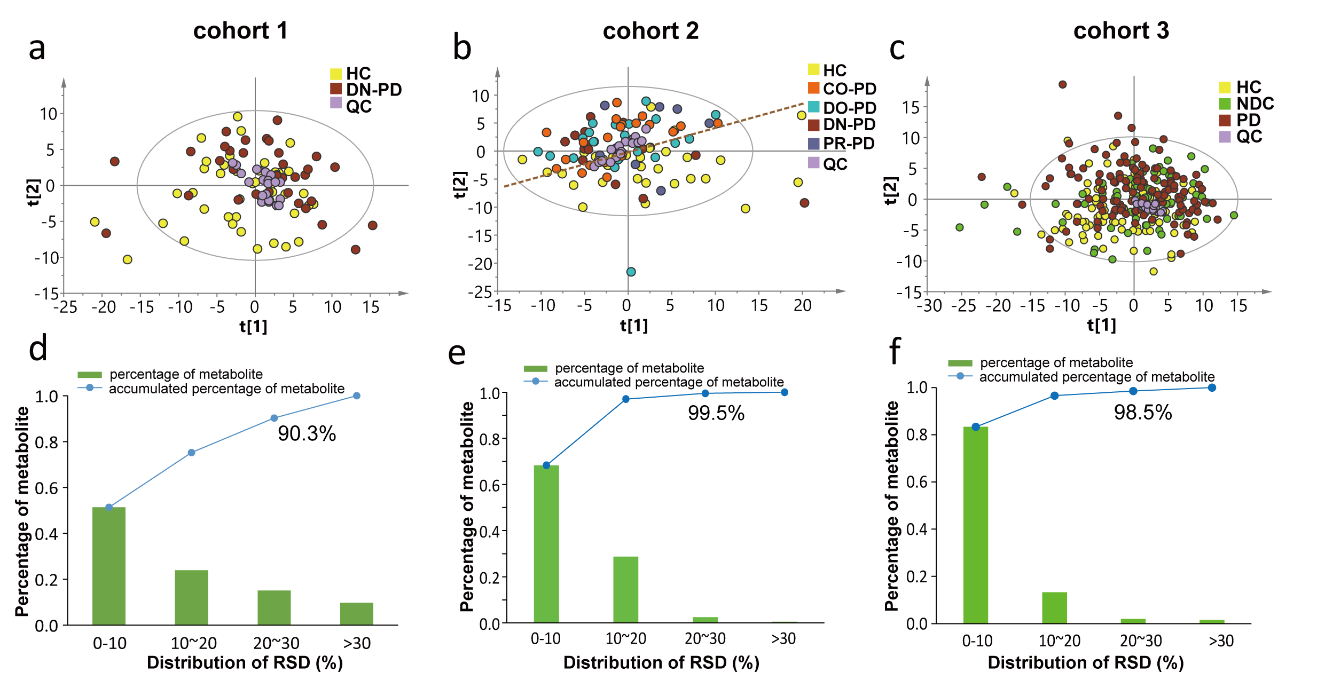


**Figure S1. Robust assessment of the analytical method across three independent cohorts. a.** Score plot of PCA in cohort 1. R2X=0.245, Q2=0.167. **b.** Score plot of PCA in cohort 2. R2X=0.269, Q2=0.186. **c.** Score plot of PCA in cohort 3. R2X=0.266, Q2=0.238. **d ~ f.** RSD distribution of metabolites in QC samples in cohort 1, cohort 2 and cohort 3, respectively.


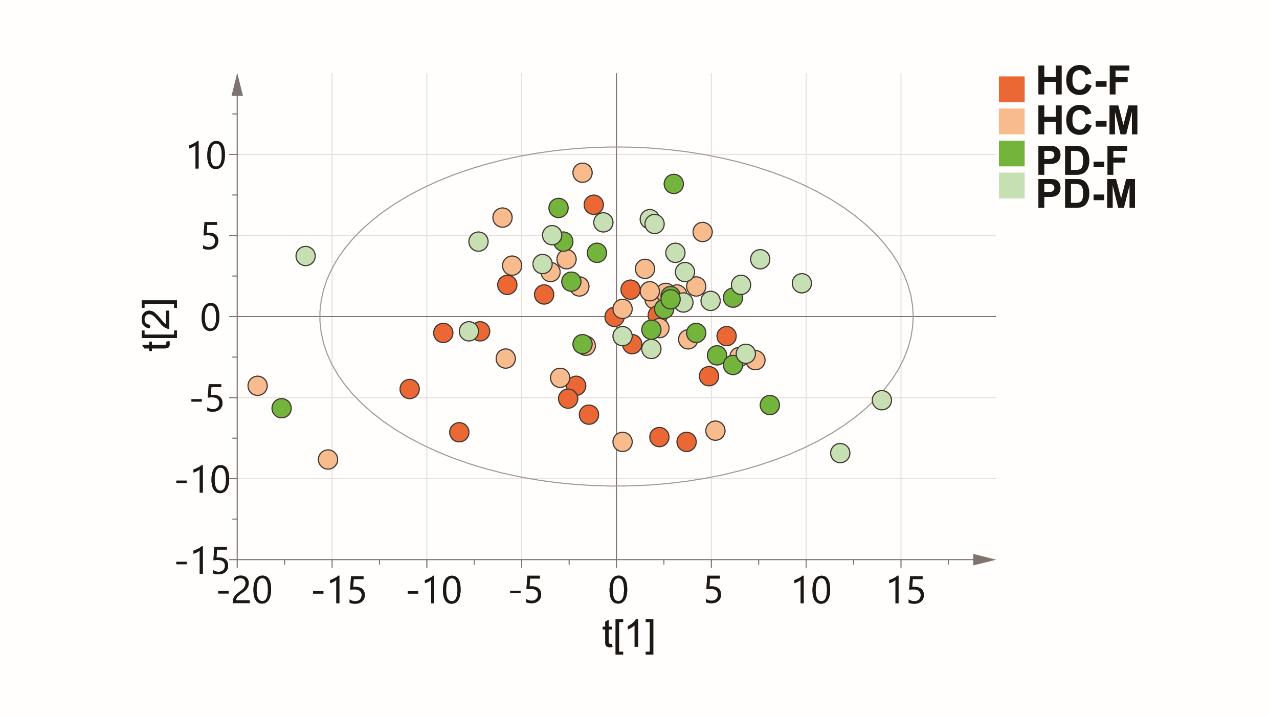


**Figure S2. PCA analysis of the metabolic profiles in** **male and female of drug-naïve PD and HC.** R2X=0.248, Q2=0.165. HC-F: female in HC; HC-M: male in HC; PD-F: female in PD; PD-M: male in PD.


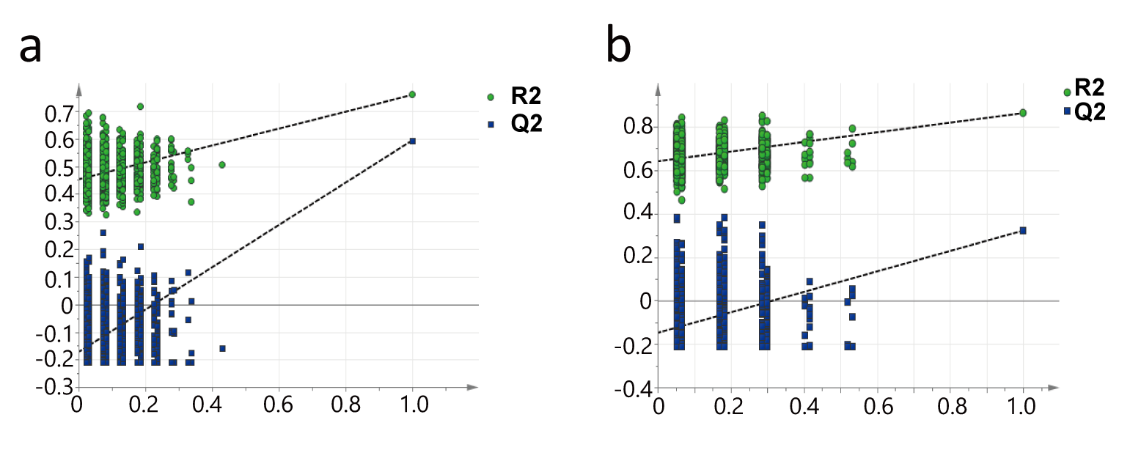


**Figure S3. Permutation test (999 times) of the PLS-DA models. a.** PLS-DA model in Figure 3a. R2=0.454, Q2=-0.164. **b.** PLS-DA model in Figure 4a. R2=0.641, Q2=-0.147.


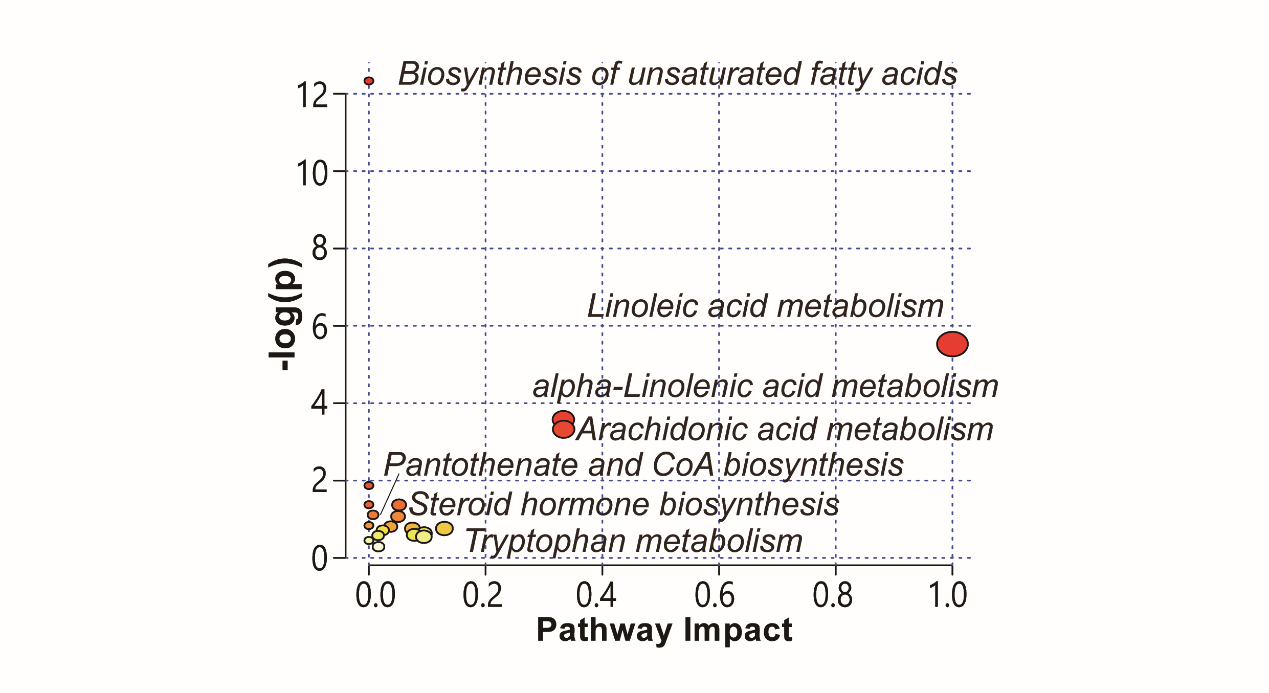


**Figure S4. Pathway analysis of the differential metabolites in drug-naïve PD compared with HC.**


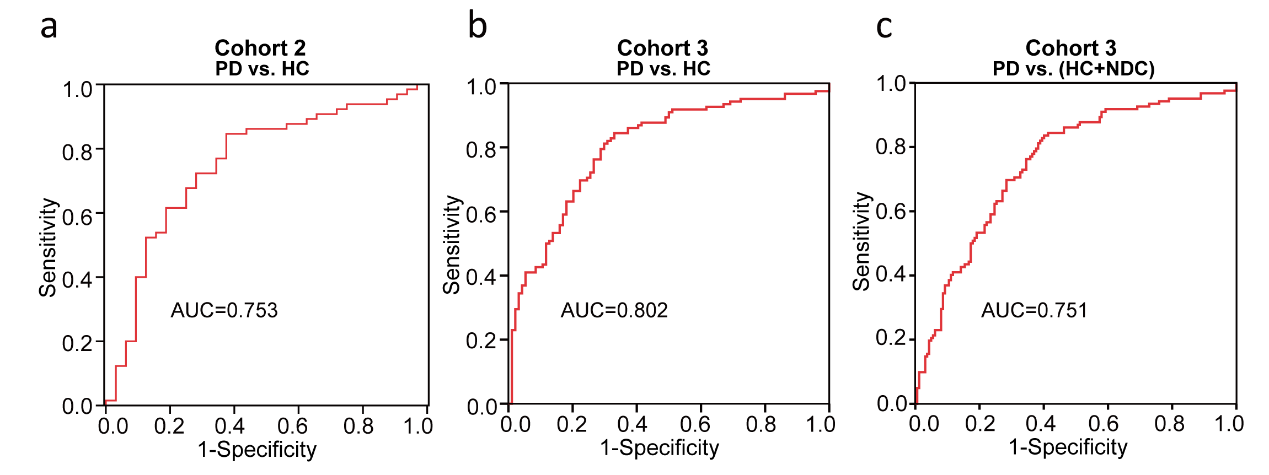


**Figure S5. The ROC curves of the metabolite panel to discriminate PD from control groups across different cohorts based on the regression equation developed in cohort 1. a.** PD vs. HC in cohort 2. The AUC value ranges from 0.647 to 0.860 at 95% CI. **b.** PD vs. HC in cohort 3. The AUC value ranges from 0.743 to 0.862 at 95% CI. **c.** PD vs. (HC+NDC) in cohort 3. The AUC value ranges from 0.693 to 0.808 at 95% CI.

**References**

1. Ren S, Shao Y, Zhao X, et al. Integration of Metabolomics and Transcriptomics Reveals Major Metabolic Pathways and Potential Biomarker Involved in Prostate Cancer. Mol Cell Proteomics 2016;15(1):154-163.

2. Ouyang Y, Tong H, Luo P, et al. A high throughput metabolomics method and its application in female serum samples in a normal menstrual cycle based on liquid chromatography-mass spectrometry. Talanta 2018;185:483-490.
